# Rab11-Rab8 cascade dynamics in primary cilia and membrane tubules

**DOI:** 10.1016/j.celrep.2024.114955

**Published:** 2024-11-08

**Authors:** Ipsita Saha, Christine Insinna, Christopher J. Westlake

**Affiliations:** 1 Laboratory of Cellular and Developmental Signaling, Center for Cancer Research, National Cancer Institute, National Institutes of Health, Frederick, MD 21702, USA; 2 Lead contact

## Abstract

The Rab11-Rab8 cascade mediated by the Rab8 guanine nucleotide exchange factor (GEF), Rabin8, orchestrates multiple membrane transport processes, but Rab membrane loading and exchange dynamics are unclear. Here, we use advanced fluorescence imaging approaches to characterize Rab11, Rab8, and Rabin8 protein dynamics. Using fluorescence ablation and recovery studies (FRAP), we show that Rab8 ciliary trafficking requires Rab11 and Rabin8. Reciprocally, we discover that Rab11 is recruited to cilia during ciliogenesis in association with Rab8. We uncover a requirement for this cascade in Rab8 association with long tubular membranes (LTMs) in human cells and zebrafish embryos. Membrane exchange dynamics of Rab11 on Rab8 LTMs is shown using super-resolution imaging, along with a dependency on Rabin8 GEF activity. Finally, cascade-dependent Rab8 loading onto enlarged Rab11-Rabin8 membrane structures is discussed. This study demonstrates that the Rab11-Rab8 cascade involves membrane conversion and expands our understanding of the cellular multifunctionality of this trafficking pathway.

## In brief

Rab11-Rab8 cascade is important in primary ciliogenesis, acute lumen formation, neurite outgrowth, and so forth. Saha et al. demonstrate, by advanced fluorescence imaging, that the Rab11-Rab8 cascade mediated by Rabin8 functions by a membrane conversion mechanism and uncover that this cascade is important for Rab8 association with membrane tubules.

## Graphical Abstract

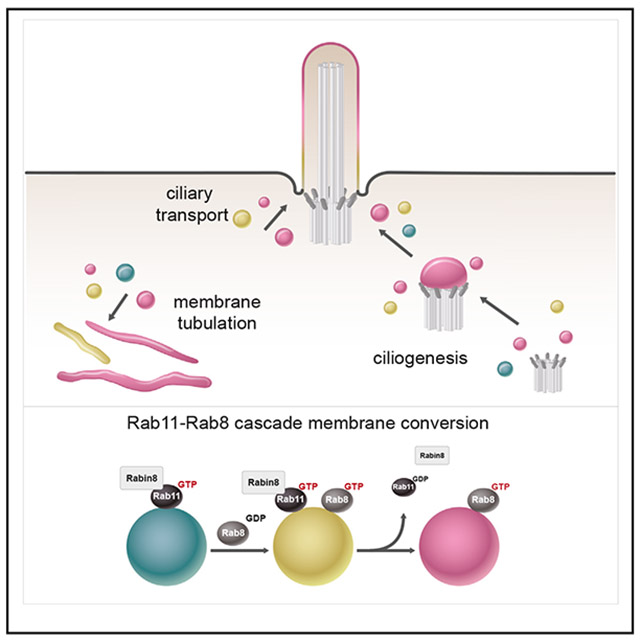

## INTRODUCTION

The Rab11-Rab8 cascade, involving the guanine nucleotide exchange factor (GEF) Rabin8 or GRAB, is important in primary ciliogenesis, neurite outgrowth, apical lumen formation, and axon outgrowth.^1-5^ This membrane trafficking pathway is highly conserved and is homologous to the yeast Rab cascade involving Ypt31/32 (Rab11)-Sec4 (Rab8) with the GEF Sec2, the Rabin8 ortholog, functioning in bud neck formation.^6,7^ In the classic Rab cascade model, an upstream active guanosine triphosphate (GTP)-bound Rab recruits a GEF to activate a guanosine diphosphate (GDP)-bound Rab functioning in the next step of membrane transport.^7-9^ Exchange of one Rab family member for another on a membrane typifies the classic Rab cascade being aided by GTPase-activating proteins (GAPs), which inactivate the upstream Rabs through catalyzing hydrolysis of GTP to GDP, leading to this Rab being chaperoned to the cytoplasm by a Rab GDP dissociation inhibitor.^10^

Rab11 and Rab8 are associated with the endocytic recycling compartment (ERC) and *trans*-Golgi network.^11-14^ Thus, a Rab cascade would be expected to enable continuous membrane trafficking in these compartments. Except for axon outgrowth, which involves GRAB,^5^ Rabin8 functions in the other reported Rab11-Rab8 cascade trafficking pathways, with both Rab8 GEFs interacting with Rab11 GTP-bound proteins.^1-4^ In the Rabin8 associated cascade mechanism, Rab11 binds to the COOH-terminal region of Rabin8, and Rabin8 activates Rab8 through its NH_2_-terminal Sec2-homology domain.^2,3^ In ciliogenesis, Rab11-dependent vesicular transport of Rabin8 to the centrioles leads to Rab8 membrane accumulation in the growing cilium.^3^ This preciliary Rabin8 vesicle trafficking also requires the Rab11 effector FIP3, which stabilizes this complex formation by also binding to Rabin8.^15,16^ A Rab11-Rab8 cascade with Rabin8 and FIP3 is also important in the ciliary transport of rhodopsin to the outer segment in photoreceptors.^17^ In neurite outgrowth, Rabin8 localizes to the Rab11 ERC, where it is thought to activate Rab8.^4^ Likewise, in cell membrane polarization, clustered small Rab11-Rabin8 and Rab11-Rab8 vesicles were observed below the developing apical lumen.^1^ Although the Rab and GEF components of Rab11-Rab8/Ypt32-Sec4 cascades have been described, it is not known whether this trafficking pathway follows a classic Rab cascade mechanism.

Microscopy imaging of Rab11-Rab8 cascade protein dynamics on cellular membranes is challenging because of the small size of the vesicles (30–50 nm in diameter), their dense cellular clustering, and rapid movement in three-dimensional space.^2-5^ In contrast, in the Rab5-Rab7 cascade, important in maturation of early endosomes to lysosomes,^11,18-21^ protein dynamics have been demonstrated on trackable large 400–800 nm endosome membrane vesicles in cells. Time-lapse imaging studies of fluorescent tagged Rab5 and Rab7 proteins show Rab5 vesicles being converted to Rab7 membranes over tens of minutes involving the Rab7 GEF Mon1-Ccz1 in human cells and in yeast with the orthologous cascade.^18-21^ Visualizing this cascade has helped expand the understanding from the basic components of Rab and GEF to the identification of other factors, including effector GEF complexes, activation feedback loops, and GAP inactivation.^11,22^ To date, this remains the only Rab cascade where the dynamics of protein loading and exchange on membranes has been visualized in cells.

Rab11 and Rab8 are known to localize to long tubular membranes (LTMs), >5 μm in length.^12,13,23^ Although the function of these structures is poorly understood, Rab8 LTMs have been linked to signaling receptor trafficking, ciliogenesis and ciliary transport, and brain cell injury response.^12,13,23,24^ Interestingly, Rab11 does not localize to Rab8 tubules,^25,26^ but Rab11 vesicles were observed along Rab8 LTMs in HeLa cells.^26^ Rab11 has also been reported to colocalize on LTMs with the Rab8 effector MICAL-L1,^27,28^ suggesting these Rabs could be linked in a cascade on these membrane structures.

Here, we investigate Rab11-Rab8 cascade protein dynamics mediated by Rabin8, associated with different cellular membrane compartments. We first tested the ciliary trafficking association of this Rab cascade using fluorescence recovery after photobleaching (FRAP) studies in cells stably expressing up to three fluorescently tagged proteins, Rab11, Rab8, and/or Rabin8. Using RNAi to deplete exogenous GFP-tagged cascade proteins, we demonstrated that Rab11 and Rabin8 cooperate to enhance Rab8 trafficking into the cilium. We also discovered that Rab11 ciliary targeting is promoted by Rab8, supporting a membrane conversion mechanism for the cascade. Next, by investigating the association of Rab8 with LTM biogenesis, we observed the colocalization of Rab8 with Rab11 and Rabin8 on these membranes, and we demonstrate a cascade dependency for Rab8 targeting to these structures. Remarkably, we detected a loss of Rabin8 and Rab11 from newly formed Rab8 LTMs using super-resolution microscopy, consistent with a membrane exchange mechanism of the GEF and upstream Rab. Requirements for Rabin8 activation of Rab8 was demonstrated in both ciliary trafficking and LTM localization using mutants affecting its GEF activity. Finally, we show loading of Rab8 onto Rab11-Rabin8 membranes by monitoring newly synthesized Rab8 proteins. Our studies reveal that the Rab11-Rab8 cascade functions via a classic Rab membrane loading and conversion mechanism.

## RESULTS

### Rab11 and Rabin8 promote Rab8 trafficking to the primary cilium

**Figure 1.**
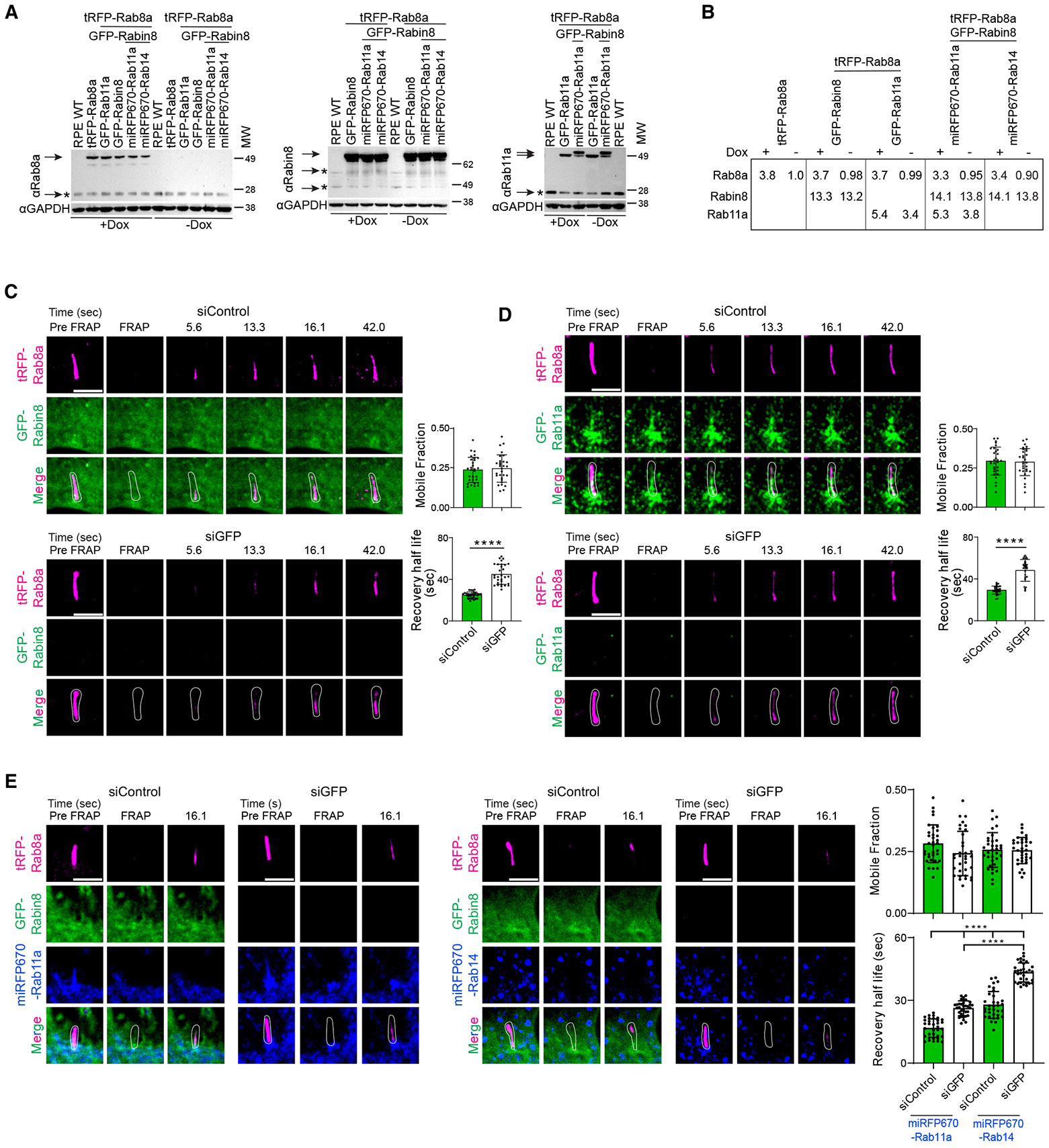
Rab8 ciliary trafficking is promoted by Rabin8 and Rab11 (A) Immunoblot analysis comparing lysates from RPE WT, tRFP-Rab8a, tRFP-Rab8a+GFP-Rab11a, tRFP-Rab8a+GFP-Rabin8, tRFP-Rab8a+GFP-Rabin8+miRFP-Rab11a, tRFP-Rab8a+GFP-Rabin8+miRFP-Rab14 cells ± Dox probed with Rab8a antibody; RPE WT, tRFP-Rab8a+GFP-Rabin8, tRFP-Rab8a+GFP-Rabin8+miRFP-Rab11a, tRFP-Rab8a+GFP-Rabin8+miRFP-Rab14, cells ± Dox probed with Rabin8 antibody; and RPE WT, tRFP-Rab8a+GFP-Rab11a, tRFP-Rab8a+GFP-Rabin8+miRFP-Rab11a cells ± Dox and probed with Rab11a antibody. Glyceraldehyde 3-phosphate dehydrogenase (GAPDH) antibody was used as a loading control. * corresponds to the endogenous protein detected in RPE WT cells and the arrows indicates the endogenous and exogenous levels in established fluorescent-tagged RPE cells. (B) Table showing densitometry of Rab11a, Rab8a, and Rabin8 proteins corresponding to arrows in (A) in ±Dox-treated cell lines compared to endogenous proteins in RPE WT cells corresponding to * in (A). Total Rab11a, Rab8a, and Rabin8 expression was normalized to GAPDH levels. (C–E) FRAP studies of ciliary tRFP-Rab8 in cells, treated with Dox for 24 h, with and without exogenous Rab11, Rabin8, and/or Rab14. Representative images of FRAP analysis performed on cilia (~5 μm) in cells treated with siControl or siGFP for 72 h, with the last 12 h serum starved to induce ciliation. Plots show the quantification of mobile fraction and half-life for the recovery of tRFP-Rab8a. (C) RPE tRFP-Rab8a+GFP-Rabin8 cells. (D) RPE tRFP-Rab8a+GFP-Rab11a cells. (E) RPE tRFP-Rab8a+GFP-Rabin8+miRFP670-Rab11a/miRFP670-Rab14 cells. Representative images from 10 cells analyzed and plots show mean ± SD from *n* = 3 experiments. ****p < 0.00001. Scale bar: 5 μm. See also Figure S1.

Rab cascade membrane dynamics can be investigated using fluorescent fusion proteins and time-lapse imaging.^18-21^ GFP-Rab8a was previously shown to traffic into the cilium following ciliogenesis by FRAP,^3^ raising the possibility that the Rab11-Rab8 cascade could be associated with Rab8 ciliary trafficking. Because Rab11a, Rab11b, and Rabin8^3,29^ are required for ciliogenesis, we devised a fluorescent protein expression approach to investigate the effects of these proteins on Rab8a ciliary trafficking in hTERT RPE1 (RPE) cells, where RNAi is used to ablate exogenous proteins without affecting endogenous protein needed for ciliogenesis. First, RPE cells were infected with lentivirus containing a doxycycline (Dox)-inducible tag red fluorescent protein (tRFP)-Rab8a and subjected to puromycin selection followed by single-cell cloning. A clonal cell line was established where the tRFP-Rab8a expression was cytomegalovirus (CMV) driven following 24-h Dox treatment and total Rab8a (tRFP tagged and untagged) was observed to be ~3.8-fold higher than observed in RPE wild-type (WT) and no-Dox-treated RPE tRFP-Rab8a cells (Figures 1A and 1B). Consistent with previous reports,^3,30^ distinctively higher ciliary localization of tRFP-Rab8a was observed compared to the rest of the cell in live and paraformaldehyde-fixed cells using spinning disk confocal microscopy (Figures S1A and S1B). FRAP analysis of this tRFP-Rab8a cell line showed a partial recovery based on determination of the mobile fraction (0.26 ± 0.07), indicative of reduced Rab8a ciliary trafficking in cells post-ciliogenesis, and a recovery half-life of 50.5 ± 9.6 s (Figure S1B). These results are consistent with our previous report using a GFP-Rab8a RPE cell line.^3^ Next, the RPE tRFP-Rab8a cells were infected with lentivirus containing the EF1α promoter to drive GFP-Rabin8 or GFP-Rab11a expression. Following blasticidine selection, RPE tRFP-Rab8a+GFP-Rab11a clonal lines expressing total Rab11a (GFP tagged and untagged) 5.4 and 5.3 times the endogenous protein in RPE WT cells were established with and without Dox, respectively (Figures 1A and 1B). For RPE tRFP-Rab8a+GFP-Rabin8, following blasticidine selection, the cells were subjected to fluorescence-activated cell sorting (FACS), and a cell line was established where total Rabin8 (GFP tagged and untagged) expressed ~13 times higher than the endogenous protein in RPE WT cells (Figures 1A and 1B). Levels of cascade proteins observed in RPE WT cells (denoted by asterisk in Figure 1A) were unaffected in fluorescent-tagged expressing cells (>0.9-fold expressed). To investigate whether GFP-Rabin8 expression affected tRFP-Rab8a ciliary trafficking, we performed FRAP studies in cells treated with small interfering (si)Control and siGFP. tRFP-Rab8a had a recovery half-life of 25.0 ± 2.9 s with siControl, whereas siGFP-treated cells were significantly slower at 44.8 ± 9.6 s, while the mobile fractions were unaffected (Figure 1C and S1C). This result demonstrates that GFP-Rabin8 promotes trafficking of tRFP-Rab8a into cilia, presumably by enhancing Rab8 activation. We next tested the effects of GFP-Rab11a on tRFP-Rab8a ciliary trafficking. Like Rabin8-expressing cells, the recovery half-life of tRFP-Rab8a was significantly faster in siControl-treated cells (29.3 ± 3.8 s) as compared to siGFP (48.2 ± 10.4 s) to ablate GFP-Rab11a, with no difference in mobile fractions being observed (Figures 1D and S1D). These findings indicate that GFP-Rab11a promotes tRFP-Rab8a ciliary trafficking. To further examine whether the effects of Rab11a and Rabin8 on Rab8a ciliary trafficking are linked to the cascade, we generated triple cell lines. A far-red miRFP670-Rab11a protein expressed under the pGK promoter was introduced into RPE tRFP-Rab8a+GFP-Rabin8 cells using lentivirus and neomycin selection followed by FACS sorting to establish a uniformly expressing cell line. In this line, total Rab11a (miRFP670 tagged and untagged) was expressed ~3.5 times above the endogenous Rab11a in RPE WT cells, and total tagged and untagged Rab8a and Rabin8 protein levels were maintained, expressing 3.3 and 14.1 times above endogenous levels of RPE WT, respectively, after Dox treatment (Figures 1A and 1B). As a control, we similarly generated a triple cell line expressing miRFP670-Rab14, a Rab that does not bind Rabin8,^3^ at levels similar to those of miRFP670-Rab11a (Figure S1E) without affecting tRFP-Rab8a and GFP-Rabin8 protein expression (Figures 1A and 1B). Significantly faster ciliary recovery of tRFP-Rab8a was observed in siControl-treated miRFP670-Rab11a-expressing cells (16.7 ± 4.4 s) compared to cells depleted of GFP-Rabin8 (26.2 ± 3.6 s), which showed comparable half-life recovery with siControl-treated Rab14-expressing cells (27.8 ± 6.4 s) (Figures 1E and S1E). However, ablation of GFP-Rabin8 significantly increased the half-life recovery of tRFP-Rab8a in the Rab14-expressing cells (43.3 ± 4.6 s). No differences in the mobile fraction of FRAP recovery under these conditions were observed. These findings support a cooperative enhancement of Rab8 ciliary trafficking by Rab11 and Rabin8 via the Rab cascade mechanism.

### Rab8 promotes Rab11 localization in primary cilia

**Figure 2.**
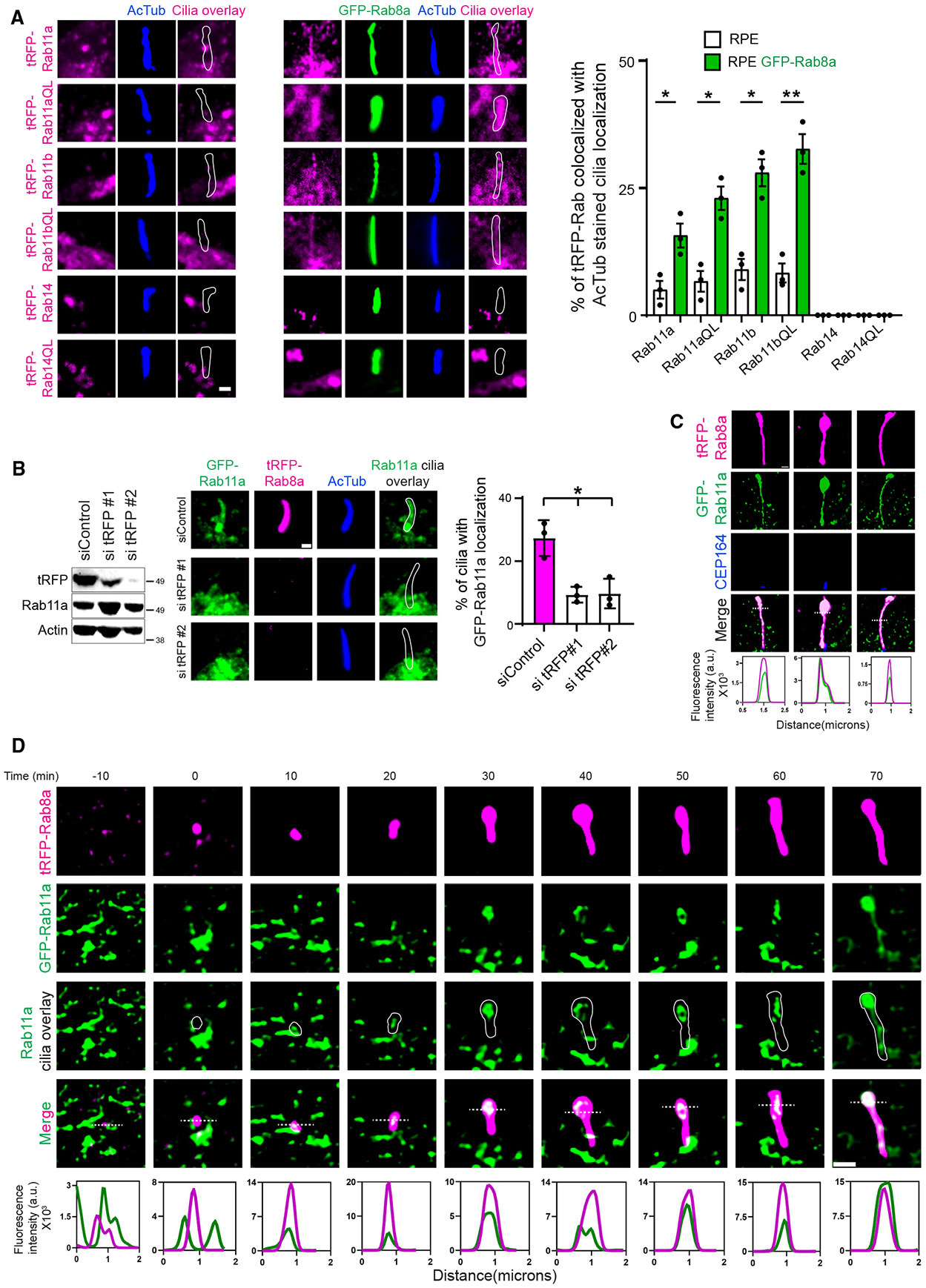
The Rab11-Rab8 cascade promotes Rab11 ciliary accumulation (A and B) Quantification of Rab8 effects on Rab11 ciliary localization. (A) Representative images of cilia in RPE (left) and RPE GFP-Rab8a (center) cells transiently transfected with tRFP-Rab WT and constitutively active (QL) proteins. (Right) Quantification of ciliary localization of tRFP-Rabs from cells starved for the last 12 h and stained with acetylated α-tubulin (^Ac^Tub) antibody showing mean ± SEM from >80 cells from *n* = 3 experiments. (B) RPE tRFP-Rab8a+GFP-Rab11a cells were treated with siControl and sitRFP for 72 h and Dox for 24 h. (Left) Immunoblot probed with tRFP, Rab11a, and actin antibodies. (Center and right) Cells were serum starved the last 12 h and stained with ^Ac^Tub antibodies. GFP-Rab11a ciliary localization was detected by spinning disk confocal microscopy (SDCM). (Center) Representative images of ciliated cells from a single xy-plane of a z stack. (Right) Quantification of ciliary GFP-Rab11a showing mean ± SEM for ~100 cells analyzed from *n* = 3 independent experiments. (C) Super-resolution imaging of Rab11 ciliary localization by Elyra 7 microscopy SIM^2^ in fixed RPE tRFP-Rab8a+GFP-Rab11a cells, treated with Dox for 24 h, serum starved the last 12 h, and stained with CEP164 antibody. Images shown are from a single xy-plane from a z stack. The lower image shows the fluorescence line profile plots of tRFP-Rab8a and GFP-Rab11a corresponding to the dotted line. (D) Super-resolution time-lapse imaging of Rab11 and Rab8 localization during ciliogenesis. Cells described in (B) were Dox treated for 24 h, serum starved for 3 h ,and then imaged live using the Elyra 7 microscope SIM^2^ every 10 min. Images shown are from a single xy-plane from a z stack. The lower image shows the fluorescence line profile plots as described in (C). Cilia are outlined in white. *p < 0.05; **p < 0.001. Scale bar: 1 μm.

We noticed that GFP-Rab11a-expressing cells display a ciliary-associated localization in cells containing tRFP-Rab8a (Figure 1D). This was also observed to a lesser extent for GFP-Rabin8 in triple exogenous lines expressing miRFP670-Rab11a (Figure 1E). Ciliary Rab11a or Rab11b and Rabin8 have not been previously described in RPE cells,^2,3^ suggesting that this localization is associated with Rab8 expression, and in the case of Rabin8, is promoted by Rab11 and Rab8. Thus, we investigated this interdependency and found that transient expression of both tRFP-Rab11a and tRFP-Rab11b showed significantly more cilia-related localization in RPE GFP-Rab8a cells than control RPE cells (Figure 2A). Rab11 localization may be linked to its activated state as the constitutively active tRFP-Rab11Q67L transiently expressed mutant had higher levels of ciliary-associated localization. These results are specific for Rab11 as tRFP-Rab14 WT and Q70L mutants were not detected in cilia. To determine whether Rab11a ciliary localization depends on Rab8, we performed RNAi studies in RPE GFP-Rab11a+tRFP-Rab8a cells and showed that treating cells with tRFP siRNA, to ablate tRFP-Rab8a without affecting endogenous Rab8a and ciliogenesis, reduced ciliary GFP-Rab11a significantly compared to siControl-treated cells (Figure 2B).

To determine whether GFP-Rab11a is in the cilium versus the ciliary pocket membrane, we performed super-resolution microscopy imaging (Figure 2C).^30^ Fluorescence line profiles confirmed that GFP-Rab11a localizes in the cilium with tRFP-Rab8a. We hypothesized that Rab11 is transported into the cilium along with Rab8 as part of the membrane conversion mechanism of a classic Rab cascade. Because exogenous Rab11 levels in mature cilia were not sufficiently high among cells, we were unable to perform FRAP studies to examine the dynamic trafficking of Rab11 in the cilium and evaluate its dependence on Rabin8 and Rab8. Instead, we tested whether dynamic Rab11 and Rab8 ciliary localization could be observed during cilium assembly using simultaneous two-color super-resolution time-lapse live cell imaging. Remarkably, we show GFP-Rab11a localizes with tRFP-Rab8a in the developing cilium (Figure 2D), a result consistent with these proteins occupying a common membrane compartment during ciliogenesis. These results support a Rab cascade mechanism whereby Rab11 membranes are converted into Rab8 ciliary membranes.

### Rab8 association with LTM requires ciliogenesis factors associated with the Rab11-Rab8 cascade

**Figure 3.**
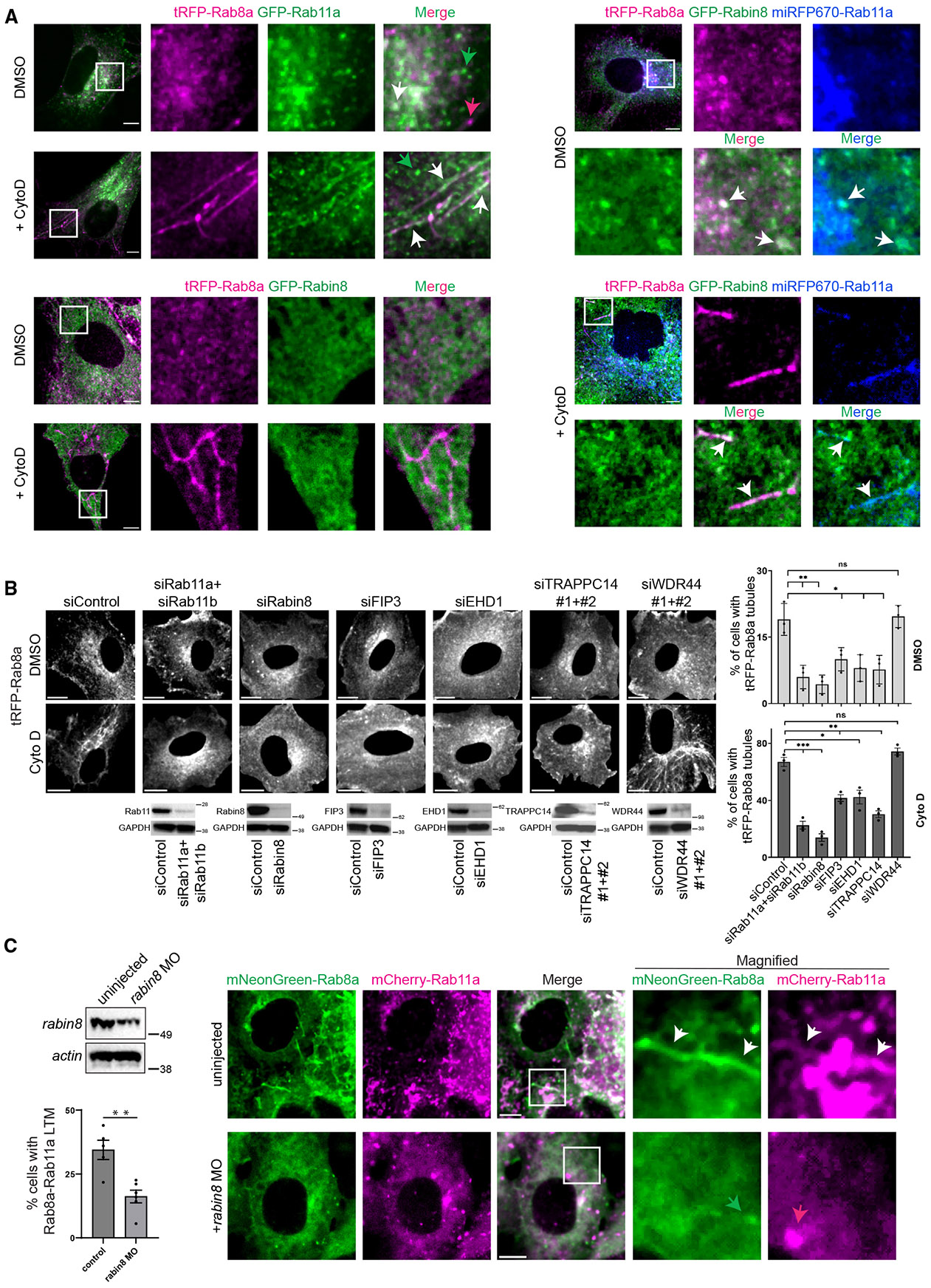
Rab11-Rab8 cascade association with Rab8 LTMs (A) Representative images of RPE tRFP-Rab8a+GFP-Rab11a, tRFP-Rab8a+GFP-Rabin8, and tRFP-Rab8a+GFP-Rabin8+miRFP670-Rab11a cells treated with Dox for 24 h and DMSO or CytoD for 30 min and imaged on an SDCM. Images shown are from a single xy-plane from a z stack. White arrows indicate colocalized vesicles or LTMs; green and magenta arrows indicate Rab11a and Rab8a vesicles that do not colocalize, respectively. (B) Quantification of tRFP-Rab8a LTMs in cells treated with siRNA for 72 h and Dox for the last 24 h, followed by DMSO or CytoD treatment for 30 min and imaged on SDCM. (Left) Representative single xy-plane image from a z stack of RPE tRFP-Rab8a treated with siRNA. (Right) Plot showing tRFP-Rab8a LTMs in cells treated with siRNA. Immunoblots (below immunofluorescence images) show protein expression from cell lysates stained with antibodies detecting actin and ablated proteins. Plot shows mean ± SEM for >100 cells from *n* = 3 independent experiments. (C) Characterization of Rabin8 dependence on Rab11-Rab8 colocalization on LTMs in zebrafish embryos. Immunoblot analysis (top left) of Rabin8 expression in 24 hpf MO-injected zebrafish embryos. Quantification of mNeonGreen-Rab8a+mCherry-Rab11a LTMs in embryos injected with mNeonGreen-Rab8a+mCherry-Rab11a with and without *rabin8* MO (bottom left). Mean ± SEM for ~100 cells analyzed per condition from *n* = 5 and 6 independent experiments for uninjected and rabin8 MO, respectively. (Right) Representative images captured on an SDCM is shown. White arrows indicate colocalized LTMs; green and magenta arrows indicate Rab8a and Rab11a vesicles that do not colocalize, respectively. ns, not significant; *p < 0.05; **p < 0.001; ***p < 0.0001. Scale bar: 5 μm. See also Figures S2-S4, Video S1, and Video S2.

Because Rab11 and Rab8 are associated with LTMs, we investigated whether the Rab11-Rab8 cascade is linked to these membrane structures. To characterize cascade factor localization in forming LTMs, we treated cells with cytochalasin D (CytoD), an actin-depolymerizing agent, which promotes acute Rab8 association with forming LTMs.^24,25,31,32^ In RPE cells stably expressing GFP-Rab11a and tRFP-Rab8a, we found that these proteins partially colocalized on LTMs following 30 min CytoD treatment, unlike DMSO-treated cells, which displayed some colocalization on small vesicles (Figure 3A; Videos S1 and S2). Similar to its ciliary localization, GFP-Rabin8 was detected on CytoD-induced LTMs when both tRFP-Rab8a and miRFP670-Rab11a were expressed, but not in cells expressing only tRFP-Rab8a or the triple line containing miRFP670-Rab14 (Figures 3A and S2), suggesting a specific requirement for Rab11. These results demonstrate that Rab11, Rabin8, and Rab8 are associated with the same dynamic LTMs. To determine whether CytoD-induced tRFP-Rab8a LTMs require Rab11 and Rabin8, we performed RNAi studies (Figure 3B). Compared to siControl-treated cells, RNAi ablation of Rab11a+Rab11b or Rabin8 significantly reduced the number of cells containing tRFP-Rab8a LTMs. This result was also observed in control DMSO-treated cells, demonstrating that the effect on Rab8 LTM localization is not dependent on CytoD. These RNAi studies indicate Rab11 and Rabin8 are required for tRFP-Rab8a association with LTMs, and moreover show that exogenous expression of Rab8 alone is insufficient to target by itself to LTMs. Rab11 requirements were further confirmed by demonstrating dominant-negative GFP-Rab11aS25N or GFP-Rab11bS25N reduced tRFP-Rab8a-positive LTMs, while other Rab dominant-negative proteins tested (GFP-Rab5 S34N and GFP-Rab14 S25N) and GFP had no effect (Figure S3). Together, these results confirm that the Rab11-Rab8 cascade is important for Rab8 LTM association.

We next considered whether other factors linked to ciliogenesis and the Rab11-Rab8 cascade function in Rab8 localization to LTMs.^16,30,33^ siRNA-depleting oligos targeting FIP3, EHD1, and TRAPPC14 significantly reduced the number of cells with tRFP-Rab8a LTMs compared to the siControl in cells treated with DMSO and CytoD (Figure 3B). In contrast, WDR44, known to prevent Rab11-Rabin8-FIP3 complex formation in ciliogenesis, did not significantly affect Rab8 LTMs (Figure 3B). These observations indicate that factors important for cascade-related ciliogenesis mechanisms also function in Rab8 association with LTMs.

Studies using zebrafish embryos have shown the Rab11-Rab8 cascade is important for ciliogenesis.^3,16,34^ To determine whether this Rab-trafficking mechanism is associated with LTMs *in vivo*, we ectopically expressed mNeonGreen-Rab8a in zebrafish embryos. At 24 hours post-fertilization (hpf), we found dynamic mNeonGreen-Rab8a LTMs in epidermal cells on the surface of the yolk sack (Figure S4). When mNeonGreen-Rab8a and mCherry-Rab11a were co-expressed, both were found to colocalize partially on vesicles and LTMs (Figure 3C). To examine requirements for Rabin8 in Rab8a/Rab11a LTM association, we injected a morpholino (MO) that depleted *rabin8* (Figure 3C). Quantification of mNeonGreen-Rab8a/mCherry-Rab11a LTMs revealed that *rabin8* depletion significantly reduced cells with these structures, supporting a requirement for the GEF in this process as observed in human cells (Figure 3B). Thus, our findings suggest that the Rab11-Rab8 cascade is associated with LTMs in cultured mammalian cells and zebrafish.

### Rab11 and Rabin8 are exchanged from newly forming Rab8 LTMs

**Figure 4.**
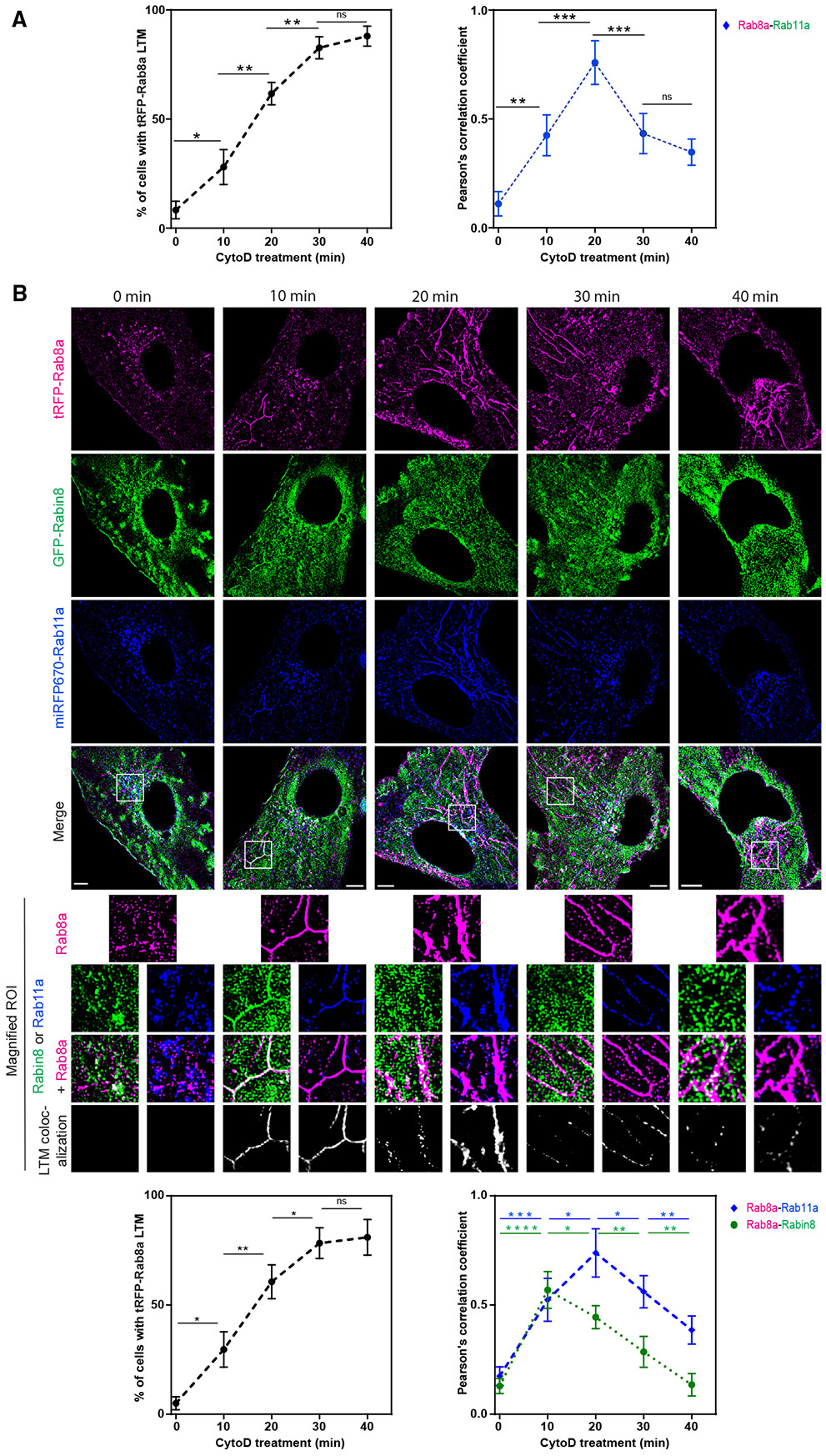
Membrane conversion protein dynamics of Rab11 and Rabin8 on Rab8 LTMs (A) (Left) Quantification of tRFP-Rab8a LTMs in RPE tRFP-Rab8a+GFP-Rab11a cells treated with Dox for 24 h (mean ± SEM for ~100 cells from *n* = 3 experiments) imaged by Elyra 7 SIM^2^ (images in Figure S5). (Right) tRFP-Rab8a colocalization with GFP-Rab11a on LTMs (calculated from Pearson’s correlation coefficient). Mean ± SEM from *n* = 3 experiments where Pearson’s correlation coefficient was calculated from 3 areas of each cell with 5 cells analyzed per experiment. (B) Representative images of RPE tRFP-Rab8a+GFP-Rabin8+miRFP670-Rab11a treated with Dox for 24 h followed by CytoD and imaged on Elyra 7 SIM^2^. tRFP-Rab8a+GFP-Rabin8 and tRFP-Rab8a+miRFP670-Rab11a colocalization on LTMs from the magnified regions of interest are shown below. Bottom images: (left plot) quantification of tRFP-Rab8a LTMs, (right plot) Pearson’s correlation coefficient for tRFP-Rab8a colocalization with GFP-Rabin8 or miRFP670-Rab11a on LTMs as described in (A). *p < 0.05; **p < 0.001; ***p < 0.0001; ****p < 0.00001. Scale bar: 5 μm.

To investigate the membrane exchange dynamics of the Rab11-Rab8 cascade during LTM assembly over time, we performed super-resolution imaging of RPE cells stably expressing tRFP-Rab8a and GFP-Rab11a (Figures 4A and S5). CytoD induced progressive organization of Rab8 into LTMs over the first 30 min after treatment^25^ and LTMs were detected in ~85% cells by 40 min. The colocalization of GFP-Rab11a and tRFP-Rab8a on LTMs was strongest at 20 min, as confirmed by determination of the Pearson’s correlation coefficients (Figures 4A and S5). Remarkably, following this time interval, there was a marked decrease in GFP-Rab11a on tRFP-Rab8a LTMs. Therefore, we can conclude that GFP-Rab11a leaves LTMs after Rab8 accumulates on these assembling membranes, suggesting an exchange occurs from Rab11 to Rab8 membranes. Next, we examined the dynamics of Rabin8 localization to LTMs using the triple cell line. Maximum colocalization of GFP-Rabin8 on tRFP-Rab8a LTMs was observed within ~10 min of their formation and progressively reduced over the last 30 min (Figure 4B). Together, these studies support a membrane conversion mechanism for the Rab11-Rabin8-Rab8 cascade organizing LTMs.

### Rabin8 GEF activity is required for Rab8 LTM association and ciliary trafficking

**Figure 5.**
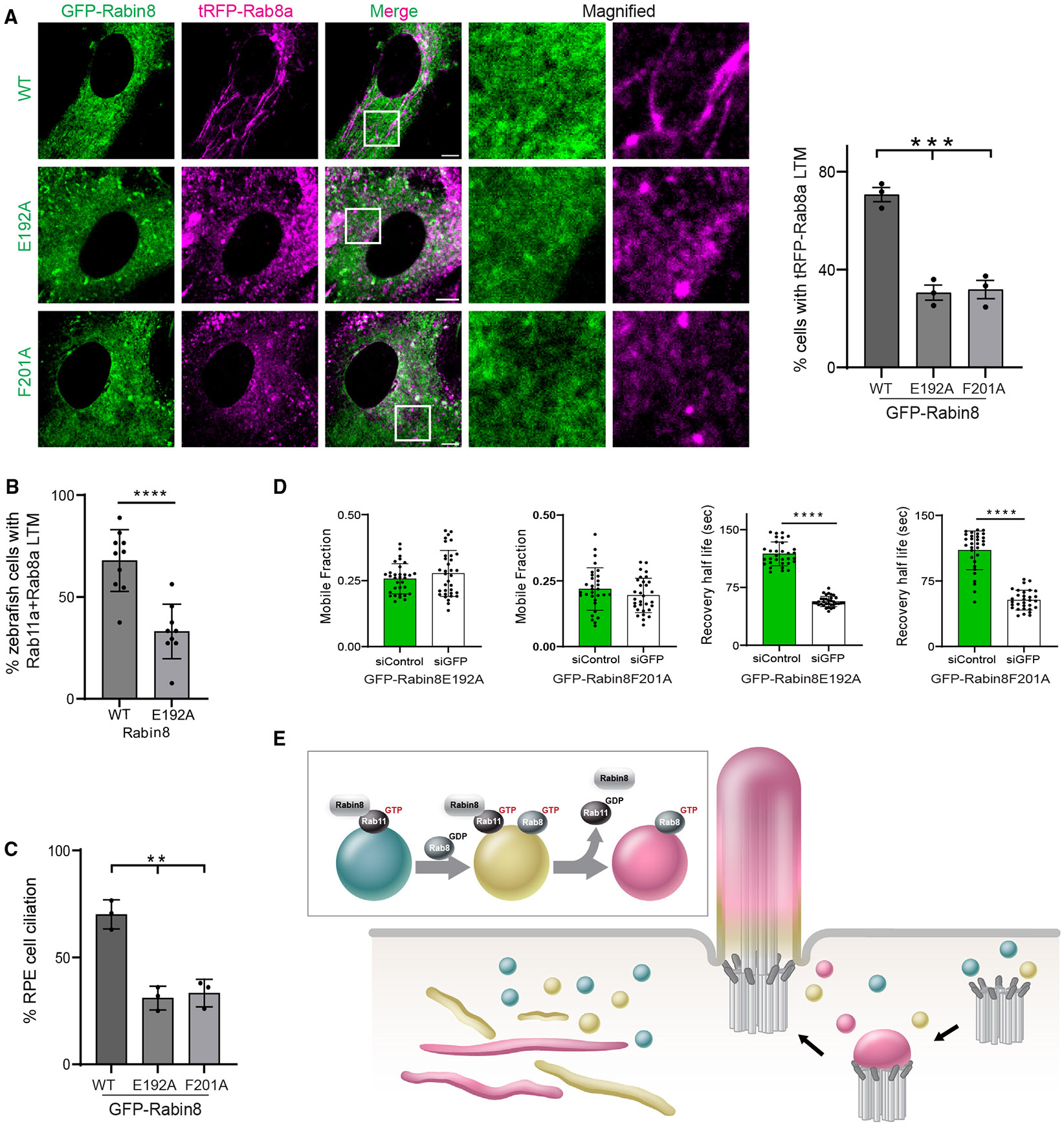
Rabin8 GEF activity is required for Rab8 LTM localization, ciliary trafficking, and ciliogenesis (A) (Right) Quantification of tRFP-positive LTMs in fixed RPE cells stably expressing tRFP-Rab8a and GFP-Rabin8 WT or GEF mutants (E192A and F201A) treated with Dox for 24 h, followed by CytoD for 30 min. (Left) Representative images from a single xy-plane of a z stack captured on an SDCM. Mean ± SEM for ~100 cells from *n* = 3 experiments. (B) Quantification of mNeonGreen-Rab8a and mCherry-Rab11a colocalized on LTMs in yolk sac cells from 24 hfp zebrafish embryos co-injected with Rabin8 or Rabin8 E192A mRNA. Mean ± SEM for *n* = 10 (Rabin8) and *n* = 9 (Rabin8 E192A) independent experiments. (C) Quantification of ciliation in cells described in (A) following serum starvation for 24 h and stained with ^Ac^Tub. Mean ± SEM for ~100 cells from *n* = 3 experiments. (D) Quantification of FRAP recovery half-life and mobile fraction performed on cilia (~5 μm) in RPE tRFP-Rab8a+GFP-Rabin8 E192A and RPE tRFP-Rab8a+GFP-Rabin8 F201A cells treated with siControl or siGFP for 72 h with Dox treatment and serum starvation for the last 24 h and 12 h, respectively. Mean ± SD from *n* = 3 independent experiments. (E) Model of Rab11-Rab8 cascade membrane conversion meditated by Rabin8. *p < 0.05; **p < 0.001; ***p < 0.0001; ****p < 0.00001. Scale bar: 5 μm. See also Figure S6.

To determine whether Rab11, Rab8, and Rabin8 dynamics observed during LTM formation depend on Rabin8 GDP-GTP exchange activity, we generated tRFP-Rab8a cell lines stably expressing the Rabin8 GEF mutants Rabin8 E192A and F201A, which have been shown to disrupt GEF activity on Rab8 without affecting Rab11 binding.^35^ GFP-Rabin8 mutants expressed similar to the WT GFP-tagged protein and showed similar tRFP-Rab8a protein levels (Figure S6A). Consistent with a dominant-negative function, GFP-Rabin8 mutants significantly reduced tRFP-Rab8a LTM localization compared to the WT protein (Figure 5A). We also examined the effects of Rabin8 E192A on Rab11-Rab8 LTMs in zebrafish (Figures 5B and S6B). Expression of Rabin8 E192A showed a significant reduction in Rab8a-Rab11a LTMs compared to the WT protein. These results demonstrate that Rabin8 GEF activity is required for Rab11-Rab8 LTM association *in vivo* as observed in human cells. We also assessed whether Rabin8 mutants affect ciliation and Rab8 ciliary trafficking. GFP-Rabin8 E192A and F201A reduced ciliation compared to the WT protein (Figure 5C), consistent with Rab11-Rab8 cascade function in ciliogenesis. We performed FRAP studies in cells that ciliated to assess whether tRFP-Rab8a ciliary trafficking required Rabin8 GEF activity (Figures 5D, S6C, and S6D). The recovery half-life of tRFP-Rab8a in the cilia of siControl-treated cells expressing the GEF mutants showed a 2-fold reduction compared to siGFP-treated cells depleted from Rabin8 mutants. Thus, the Rabin8 GEF activity requirement for Rab8 localization in ciliary and LTM membranes is consistent with its function in the Rab11-Rab8 cascade (Figure 5E).

### Rab8 is loaded onto enlarged Rab11-Rabin8 vesicular membranes

**Figure 6.**
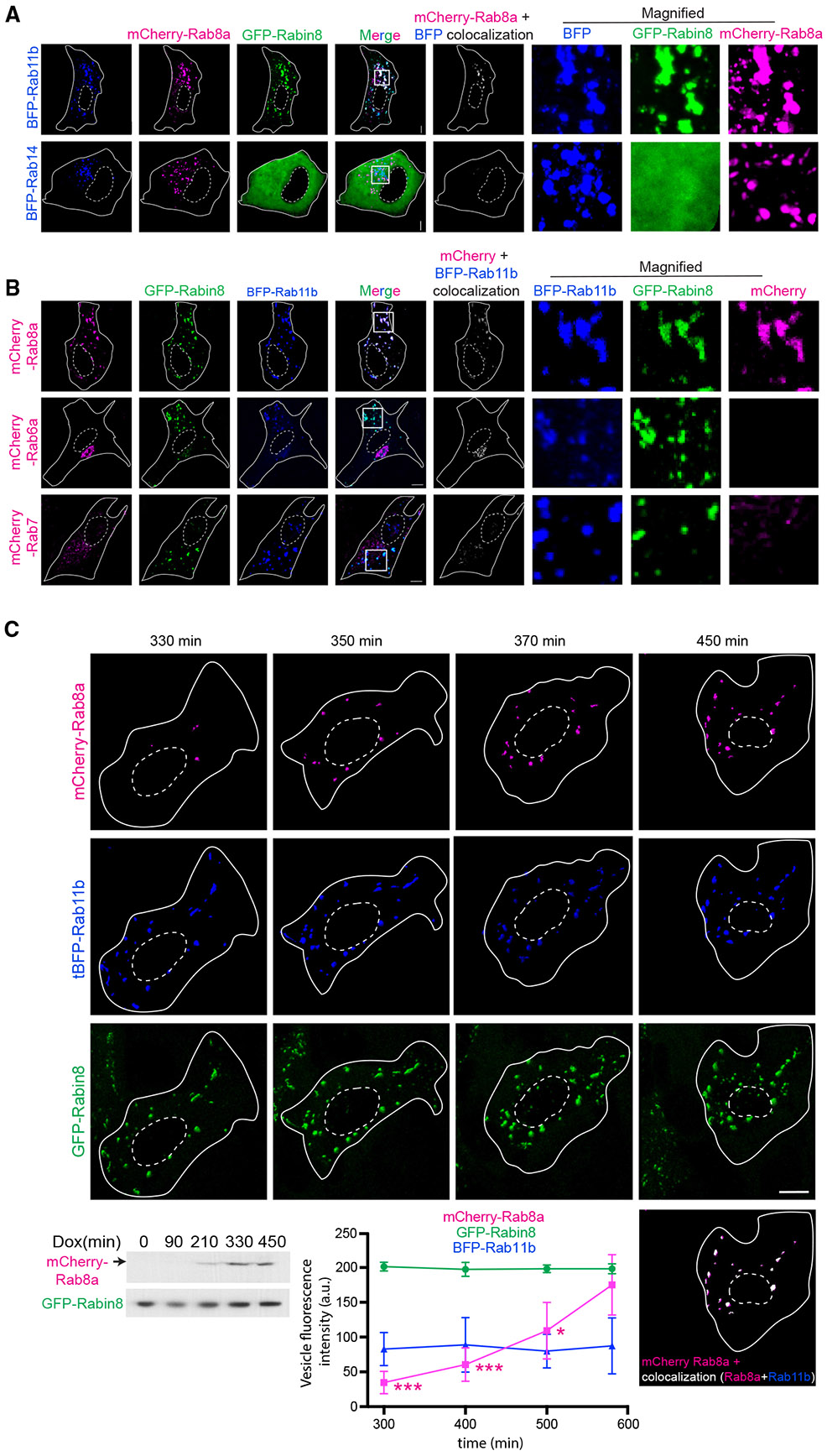
Rab8 is loaded onto enlarged Rabin8-Rab11 membranes (A) RPE cells transiently transfected with mCherry-Rab8a, GFP-Rabin8, and tBFP-Rab11b or BFP-Rab14 for 48 h. Representative images show a single xy-plane from a z stack captured on SDCM using a Hamamatsu Orca Flash4 camera. mCherry+tBFP colocalization is shown. (B) RPE GFP-Rabin8 cells transiently transfected with tBFP-Rab11b and mCherry-Rab8a, mCherry-Rab7 or mCherry-Rab6a for 48 h. Representative images show a single xy-plane from a z stack captured on SDCM with an EMCCD Evolve 512 camera. (C) RPE GFP-Rabin8+mCherry-Rab8a (Tet-inducible) cells transiently expressing tBFP-Rab11b and treated with Dox 24 h post-transfection imaged by time-lapse microscopy every 20 (representative images) or 25 min (plot). Representative images (top) show three-dimensional surface rendering of unfiltered z stack (step size, 1 μm) cell images for the time course. Image of BFP-Rab11b colocalization (white) with mCherry-Rab8a is shown at time point 450 min. Immunoblot shows expression of mCherry-Rab8a, detected with anti-Rab8 antibodies, and GFP-Rabin8, detected with GFP antibodies, following indicated Dox treatment times. Plot shows tBFP-Rab11b, mCherry-Rab8a, and GFP-Rabin8 fluorescence intensity in tBFP-positive vesicular structures (≥1 voxel) measured at the indicated times following Dox induction and plotted for five cells. Results shown as mean ± SD from two independent experiments. *p < 0.05; ***p < 0.0001. Scale bar: 5 μm.

Transiently expressed mCherry-Rab8a, GFP-Rabin8, and BFP-Rab11b cascade proteins colocalize on large vesicles in RPE cells (Figure 6A), which are of a similar size and trackability as described in studies examining Rab5-Rab7 cascade dynamics.^18^ Consequently, we investigated whether these structures could be used to examine Rab8 loading onto Rab11-Rabin8 membranes. Importantly, this vesicular co-localization was Rab11-dependent as GFP-Rabin8 remained cytoplasmic following the expression of BFP-Rab14, which localized to large vesicles that did not overlap with mCherry-Rab8a (Figure 6A). A similar result was observed in a clonal RPE GFP-Rabin8^3^ cell line transiently expressing mCherry-Rab8a and BFP-Rab11b (Figure 6B). The specificity of Rab8a association with Rab11b-Rabin8 membranes was further shown by comparing mCherry-Rab7 or mCherry-Rab6a localization, which do not accumulate on GFP-Rabin8-BFP-Rab11b membranes (Figure 6B). As with our analysis of the dynamics of Rab8 at the cilium and LTMs, it was not possible to conclude from these studies whether Rab8 accumulation on large Rab11-Rabin8 vesicles was directly related to cascade protein dynamics or resulted from membrane fusion. To address this question, we devised a cellular assay to examine the localization of newly translated Rab8 in cells containing Rabin8-Rab11b enlarged vesicles (Figure 6C). RPE GFP-Rabin8 cells were infected with a lentivirus encoding a Dox-inducible mCherry-Rab8a protein, and following puromycin selection, a clonal cell line was established. mCherry-Rab8a could be detected by immunoblotting after 210 min of Dox treatment and steadily increased over a 450-min time period (Figure 6C). Strikingly, time-lapse fluorescence imaging showed a significant increase in mCherry-Rab8a accumulation on punctate GFP-Rabin8 and BFP-Rab11b membranes in cells 300–500 min after Dox treatment. mCherry-Rab8a that was not associated with GFP-Rabin8/BFP-Rab11b membranes was diffusely distributed throughout the cytoplasm and was not observed on other trafficking vesicles. Importantly, GFP-Rabin8 and BFP-Rab11b fluorescence signal was unchanged on membranes over the course of the time-lapse imaging, indicating that the targeting of these proteins was unaffected by mCherry-Rab8a expression and was maintained on these membranes, as was expected from our results in Figure 6B. These results illustrate that newly synthesized mCherry-Rab8a is loaded onto GFP-Rabin8-BFP-Rab11b membranes consistent with a classic Rab cascade mechanism (Figure 5E).

## DISCUSSION

The Rab11-Rab8 cascade with Rabin8 has been linked to several cellular trafficking processes, yet direct demonstration of a classic Rab cascade and dynamics of these proteins, such as was demonstrated for the Rab5-Rab7 cascade, has not been reported. Here, we characterize membrane exchange dynamics of Rab11 and Rabin8 and loading of Rab8 (Figure 5E) using fluorescently tagged proteins exogenously expressed in cells at determined levels. We also demonstrate the Rab11-Rab8 cascade is required for Rab8 LTM association in human cells and zebrafish embryos, which share conservation in assembly requirements with ciliogenesis.

Challenges with studying protein dynamics on membranes, such as those associated with the Rab11-Rab8 cascade, include speed, directionality, and high density of vesicles that affect the monitoring of membrane loading and exchange events, even at a single site in the cell such as the centrioles/basal body/cilia (Figures 1 and 2D).^2-5^ Moreover, direct monitoring of Rab11-Rab8 cascade protein dynamics in developing and mature cilia is affected by the strong ciliary Rab8 signals, which effectively blind the visualization of nearby vesicles containing lower levels of the protein (Figures 1 and 2D). Investigation of the cascade dynamics on acutely forming tRFP-Rab8a LTMs by super-resolution imaging enabled characterization of the Rab11 and Rabin8 membrane exchange on these Rab8 structures. Analysis of Rab8 activation and loading onto assembling LTMs could not be evaluated using this approach due to the dynamic nature and degree of expansion of these membranes stimulated by CytoD (Video S2). Furthermore, conclusions about Rab8 activation on newly forming LTMs could not be made due to the possibility that some Rab8 was activated and loaded onto smaller vesicles that subsequently fuse with these larger membranes (Video S1). However, using an inducible expression system, newly translated Rab8 could be directly observed loading onto enlarged Rab11b-Rabin8 membranes by time-lapse imaging. Our work also supports a classic Rab cascade mechanism for Rab11 and Rab8 in developing and mature cilia. FRAP studies demonstrating that Rab11 and Rabin8 together enhance Rab8 trafficking to the mature cilium are consistent with the cascade mechanism. The fact that mobile fractions of the Rab8a recovery remain unaltered by ablation of Rabin8 and/or Rab11 suggests that the cascade is involved in ciliary entry and does not affect trafficking out of the cilium. Moreover, the discovery that Rab11 is targeted to the mature cilium in a Rab8-dependent manner and accumulates in this organelle during ciliogenesis supports a classic Rab cascade membrane exchange model where the Rab11 ciliary localization results from incomplete conversion from a Rab11 to a Rab8 compartment. The trafficking processes examined, coupled with the use of expressed fluorescent tagged proteins, enabled testing Rab11-Rab8 cascade protein membrane dynamics by live cell imaging.

It is anticipated that the approaches used to study the Rab11-Rab8 cascade will open prospects of a deeper understanding of this trafficking mechanism, similar to the Rab5-Rab7 cascade. In this cascade, a Rab5 activation feedback loop has been identified whereby Mon1-Ccz1, the Rab7 GEF, displaces the Rab5 GEF, Rabex5, from the endosomes.^19^ GEF function on Rab11 that is important for the Rab11-Rab8 cascade has not been reported. Interestingly, components of the Rab11 GEF TRAPPII (transport protein particle II) complex^36^ are important for ciliogenesis,^3,33^ including TRAPPC14, which binds to Rabin8^33^ and affects Rab8 LTM targeting (Figure 3B), suggesting that further investigation of the TRAPPII complex GEF-related function in the cascade is merited. In addition, a GAP, RabGAP5, has been shown to function in exchange of Rab5 from developing late endosomes.^37^ Our observations showing dynamic Rab11 association with ciliary and LTM structures suggest that an unknown GAP could function in converting Rab11 compartments to Rab8. Examination of Rab11-Rab8 cascade dynamics could also be used to better understand other modes of regulation linked to this pathway. Cascade-related Rabin8 GEF function has been linked to kinase regulation by Akt and NDR2, but it is not clear whether this is associated with protein membrane dynamics.^16,38^ Notably, NDR2-mediated Rabin8 phosphorylation at Ser272 promotes binding to the exocyst protein Sec15, a Rab8 effector, a step suggested to be important for retaining the GEF at the basal body during ciliogenesis.^38^ Changes in phospholipid levels accompanying membrane conversion may also affect Rab11-Rab8 cascade dynamics. Notably, Ypt32-Sec2 interaction is enhanced by Sec2 binding to phosphatidylinositol-4-phosphate, and as this phospholipid levels decrease on the membrane, Sec2 switches effector binding from Ypt32 to Sec15.^7^ A phospholipid associated feedback loop has also been proposed for the Rab5-Rab7 pathway. Rab5 recruits the PI3 kinase Vps34, which promotes the synthesis of PIP3 on early endosomes, and the resulting increased PIP3 levels help Rab5-GTP recruit Mon1-Ccz1 for Rab7 activation.^22^ Thus, Rab cascades are expected to involve additional co-factors besides the GEF and a GAP, and may be coordinated with alterations in phospholipid levels.

Further examination of Rab11-Rab8 cascade membrane dynamics may also reveal how multiple cellular processes utilize this trafficking mechanism and explain the specification of Rabin8 and GRAB GEFs. Interestingly, this Rab cascade has been linked to tunneling nanotube formation in neuronal CAD cells, but it involves a mechanism independent of the aforementioned Rab8 GEFs, suggesting that additional unknown GEFs could function in this pathway.^39^ Likewise, it is not clear whether cascade co-factors specify different Rab11-Rab8 cascade pathways. For example, Myo5B is required for apical lumen formation and has effector-based interaction with Rab11 and Rab8 and associates with LTMs,^26,40^ but its role in ciliogenesis has not been explored. Our work shows that membrane trafficking regulators linked to the Rab11-Rab8 cascade are important for ciliogenesis and Rab8 LTM association, suggesting that common regulatory mechanisms exist for cascade-related pathways. Thus, extension of the approaches used in this study to examine GEF-specific function and other regulators linked to Rab11-Rab8 cascade pathways will help define mechanistic conservation and distinctions used in different cellular processes.

LTM formation of the ERC is associated with Rab11 and Rab8, among other Rabs,^12,13,25,27,32,41^ although the Rab11-Rab8 cascade had not been previously linked to this process. However, reports show that Rabs from this cascade associate with Myo5B, MICAL-L1, and EHD1, proteins known to regulate LTMs.^13,27,40,42-44^ Our work shows that Rab11, Rabin8, and proteins (EHD1, FIP3, TRAPPC14) associated with this pathway in ciliogenesis are required for Rab8 association with LTMs in RPE cells. It is not clear whether LTMs can form without the Rab11-Rab8 cascade in cells, and notably, MICAL-L1 LTM association occurs without Rab8 in HeLa cells.^42^ It also remains to be determined whether LTMs associated with the Rab11-Rab8 cascade are important for cargo transport. Nonetheless, Rab8 colocalizes with the transferrin receptor in these membranes,^25^ and both Rab8 and Rab11 function in transferrin receptor recycling.^12,13^ Interestingly, Rab8 LTMs have been implicated in astrogliosis, a critical response to CNS cell injury response.^24^ Rab8 LTMs are also associated with developing and mature cilia, and Rab8 removal from the cilium can occur via these structures forming from the ciliary pocket membrane,^23^ but whether these LTMs are associated with Rab11-Rab8 cascade remains undetermined. Finally, our finding that the Rab11-Rab8 cascade is associated with LTMs detected in zebrafish embryos suggests that these structures could be important during development. These open-ended questions point to a need for further research into Rab11-Rab8 cascade connections to these poorly understood LTMs.

It has been more than two decades since the Ypt32-Sec4/Rab11-Rab8 cascade was first described. This study demonstrates how protein dynamics on membranes associated with the Rab11-Rab8 cascade can be investigated. Besides the Rab11-Rab8 and Rab5-Rab7, other Rabs have been proposed to utilize a classic cascade mechanism.^4,45^ Thus, with more than 60 Rabs in the human genome, this raises the possibility that many more Rab cascade combinations have yet to be discovered, and therefore a better understanding the Rab11-Rab8 cascade is expected to provide insight into this general mechanism of vesicle trafficking regulation in the cell.

### Limitations of the study

Our study demonstrates that the Rab11-Rab8 cascade, mediated by the GEF Rabin8, follows a classic membrane conversion mechanism in cultured RPE cells and zebrafish embryonic cells in different membrane compartments. Consistent with other reported Rab cascade dynamics studies, it is necessary to express exogenous Rab11a, Rab8a, and Rabin8 proteins to visualize the dynamics of this cascade in live cells. In addition, a drug, CytoD, and induction of non-dynamic enlarged Rab11-Rabin8 membranes were used to demonstrate Rab11 exchange from tubules and Rab8 membrane loading, respectively. We were unable to demonstrate the full cascade on a single membrane compartment (i.e., loading of Rab8 onto Rab11 membranes and loss of Rab11 from the newly forming Rab8 membrane compartment). Thus, future evaluation of physiological trafficking pathways associated with the Rab11-Rab8 cascade should be pursued, including those based in zebrafish, which we have shown can develop LTMs associated with this pathway. We have used advanced fluorescence imaging with Elyra SIM^2^ to demonstrate the association of Rab11, Rabin8, and Rab8 on tubules. While the lateral resolution is close to the resolution limit of the tubules (~80 nm), the axial resolution is limited to ~170 nm. Thus, higher-resolution imaging coupled with correlative light and electron microscopy could provide better details of the spatial association of these proteins. Developing new biochemical approaches that can evaluate transient GEF-based Rab-activation events on membrane compartments to examine the dynamics of this pathway and Rab cascades in general would be merited. Finally, our study demonstrates that Rab11, Rabin8, and other ciliary trafficking factors are essential for Rab8 association with LTMs, but it does not show whether these trafficking factors are essential for the formation of LTMs in RPE cells. We anticipate that exploring other markers of tubular membranes coupled with volume electron microscopy will enable investigators to address this question.

## STAR★METHODS

### EXPERIMENTAL MODEL AND STUDY PARTICIPANT DETAILS

#### Zebrafish

Experimental procedures using AB zebrafish were performed in accordance with protocols (ASP#23–416) approved by the animal care & use committee of the National Cancer Institute at Frederick in compliance with the Association for Assessment and Accreditation of Laboratory Animal Care (AAALAC) guidelines. Animals were maintained at 28°C.

#### Cell lines

##### hTERT-RPE1 cells (RPE)

Parental hTERT-RPE1 (RPE) cells were purchased from ATCC. Parental and stable expressing RPE cell lines were cultured in DMEM/F12 supplemented with 10% FBS and 1% PenStrep at 37C in 5% CO2 supply.

##### 293T

293T were purchased from ATCC and were cultured in DMEM supplemented with 10%FBS, 1%L-Glutamine and 1% PenStrep at 37C in 5% CO2 supply.

### METHOD DETAILS

#### Chemicals and reagents

Cytochalasin D was from Tocris Bioscience (#1233) and used at a final concentration of 100nM. Doxycycline monohydrate was from Sigma (#D1822) and used at a final concentration of 1μg/ml. Lipofectamine 3000 (#L3000001, Life Technologies), Fugene6 (Promega, #E2691) were used for RPE cells and Polyjet (#SL100688, Signagen Laboratories) was used for 293T cells, as transfection reagents according to the manufacturer’s instruction. Lipofectamine RNAiMAX (#13778150, Life Technologies) was used as transfection reagent for siRNA transfection according to the manufacturer’s instruction. Puromycin (10μg/ml, Invitrogen, # A1113803), Blasticidin (10μg/ml, Santa Cruz biotechnology, # sc-495389) and Neomycin (0.25 mg/ml, Invitrogen, #10131035) were used for cell selection.

#### Plasmids

Lentivirus plasmid psPAX2 (Addgene, #12260) and PMD2.G (Addgene, #12259), pENTR-Rab7 (Thermo IOH40569) (Genbank: NM_004637.6), pENTR-Rab14 (Thermo, IOH6559) (GenBank:NM_016322.4), pTagBFP-N(Evrogen #FP172) and mNeonGreen (Addgene #189976) were purchased. pENTR plasmids for Rab11a (GenBank: NM_004663.5), Rab11b (GenBank: NM_004218.4), Rabin8 (GenBank:NM_175623.4) and Rab8a (GenBank: NM_005370) have been described.^3,16,30^ Rab8a was gateway cloned using LR clonase II (Thermo Fisher Scientific, #11791100) into pHUSH-tRFP, an inducible lentivirus expression vector reengineered by PCR to have CMV promoter, TetO_2_ and tRFP tag as previously described in,^30^ to generate pHUSH tRFP-Rab8a. PCR-amplified Rab5a (GenBank: NM_004162) and Rab6a (GenBank: NM_198896) were cloned into pDONR221 plasmid using BP Clonase II (Thermo Fisher Scientific, #11789100). Rabin8 mutants (Rabin8 E192A and F201A) were generated by PCR using Phusion polymerase (#F632S, Thermo Fisher Scientific). Rab8a, Rab7 and Rab6a gateway entry clones were subcloned using LR recombination reactions into the pHUSH-mCherry plasmid, a vector generated by replacing mCherry for the LAP tag in the previously described pHUSH LAP vector^30^, to generate mCherry-Rab8a, mCherry-Rab7 and mCherry-Rab6a. Rab11b and Rab14 were PCR amplified and cloned into pDONR221 P5,P2. BFP was PCR amplified from pTagBFP-N(Evrogen #FP172) and cloned into pDONR221 P1-P5r. Multisite gateway cloning with LR Clonase II+ (#12538120, Invitrogen) was used to generate BFP-Rab11b and BFP-Rab14 fusion constructs cloned into a gateway compatible pCS2+^30^ vector with an NH_2_-terminal FLAG-TEV-Stag tag sequence. pENTR Rab11aS25N, Rab11bS25N, Rab5aS34N and Rab14S25N were generated using QuickChange II mutagenesis kit (Catalog no. 200524, Agilent Technologies) and subcloned in pDEST53 to generate GFP-Rab5aSN, GFP-Rab14SN, GFP-Rab11bSN. pENTR Rab11aQ67L, Rab11bQ67L and Rab14Q70L were generated using QuickChange II mutagenesis kit, and along with pENTR Rab11a, Rab11b and Rab14 were subcloned in pDEST-tRFP, a vector previously described in^3^, to generate tRFP-Rab11a, tRFP-Rab11aQL, tRFP-Rab11b, tRFP-Rab11bQL, tRFP-Rab14 and tRFP-Rab14QL. mNeonGreen-Rab8a, mCherry-Rab11a, Rabin8 and Rabin8E192A were subcloned into pCS2+ (Addgene, Kit#1000000107) or pCS2-GFP (#86723, Addgene). To generate pDEST686-pGK-miRFP670 Rab11 or Rab14 plasmids, pPGK (Addgene #197201), miRFP670(5,1) (obtained by cloning miRFP (Addgene, #197201) into pDONR235 using BP clonase) and Rab11a or Rab14 were subcloned into pDest 686^47^ by Multisite gateway using LR clonase II. To generate pDEST658-EF1α GFP-Rab11a or Rabin8 wildtype or mutants, EF1α (Addgene, #162975), GFP (Addgene, #162970) and Rab11a, Rabin8 wildtype and mutants were subcloned into pDest 658^47^ by Multisite Gateway using LR clonase II.

#### Cell lines

Human Retinal Pigment Epithelial cells (RPE) (hTERT-RPE1, ATCC, #CRL-4000) were cultured in DMEM/F12 supplemented with 10% FBS and 1% PenStrep. 293T (ATCC, #CRL-3216) were cultured in DMEM supplemented with 10%FBS, 1%L-Glutamine and 1% PenStrep. RPE GFP-Rab8a and GFP-Rabin8 cells have been previously described in.^3^ To generate lentivirus, the lentiviral plasmid was co-transfected with psPAX2 and PMD2.G into 239T using Polyjet (#SL100688, signagen) transfection reagent according to the manufacturer’s protocol. RPE cells were infected with lentivirus generated from pHUSH tRFP-Rab8a, and selected with puromycin and single cell cloned by limited cell dilution into 96 well plates. Rab8 protein expression in clones was compared to RPE cells by immunoblotting. A clonal RPE tRFP-Rab8a cell line was subsequently infected with lentivirus generated from EF1α GFP-Rab11a and selected with blasticidin. These cells were single cell cloned by limited cell dilution into 96 well plates. Rab11a protein levels were evaluated by immunoblotting and a clonal line was identified.

To generate RPE tRFP-Rab8a+GFP-Rabin8, RPE tRFP-Rab8a+GFP-Rabin8 E192A and RPE tRFP-Rab8a+GFP-Rabin8 F201A, the RPE tRFP-Rab8a cells were infected with virus containing EF1α GFP-Rabin8, EF1α GFP-Rabin8 E192A or EF1α GFP-Rabin8 F201A, selected with blasticidin and FACS sorted to ensure uniform expression in cells. RPE tRFP-Rab8a+GFP-Rabin8 were infected with lentivirus generated from pGK miRFP670-Rab11a or pGK miRFP670-Rab14 and then selected with Neomycin, to generate RPE tRFP-Rab8a+GFP-Rabin8+miRFP670-Rab11a or miRFP-670-Rab14. These cells were then FACS sorted to ensure uniformity in expression of proteins.

RPE GFP-Rabin8 cells^3^ were infected with lentivirus generated from the pHUSH mCherry-Rab8a and selected with puromycin as described in.^30^ All cell lines were checked periodically throughout experimentation to ensure they maintained similar exogenous expression of proteins.

#### Microscopy

For FRAP assays all cells were plated on glass bottom plates and treated with or without siRNA for 72 h with serum starvation the last 12 h to promote ciliation. The cells were imaged, at 37C in 5% CO_2_ atmosphere, using a Marianas spinning disc confocal microscope (SDCM) (Intelligent Imaging Innovations) with a 63X 1.3NA Zeiss oil objective. The images were captured using a sCMOS (Hamamatsu ORCA Flash4) camera. The cells were acclimatized in the microscope for 1 h and stabilization system (Definite focus) of the microscope was used to avoid drifting of the sample. The cilia region of interest was photobleached after 3 frames for 2 s using Vector (Intelligent Imaging Innovations) and the v561 laser. Frames were captured every 600ms for 2 channel imaging or 900ms for 3 channelimaging for a single z-plane. The channels were captured synchronously. The raw images were photobleach corrected and intensities were exported using SlideBook software. The FRAP recovery curves were fitted to the classic FRAP recovery equation $I(t)=I_{0}m(1-e^{-i\tau t})$ (where half-life $t_{1∕2}=\frac{ln\;0.5}{\tau },I_{0}$ is the normalized intensity obtained after photobleach correction and *m* is the mobile fraction) using MATLAB curve fitting tool.

For ciliation assays, the cells were serum starved 12 h followed by immunostaining, imaging and ciliation analysis as described.^23,30^ Briefly, the cells were washed with PBS, fixed using 4% PFA for 10 min, washed with PBS and then blocked for 1 h at room temperature using blocking buffer (0.5% BSA, 0.1% Triton X-100 in PBS) and then immunostained with antibodies in this blocking buffer. For ciliation imaging, 10 different ROIs were captured for each experiment on the SDCM using 63X 1.3NA Zeiss oil objective with a sCMOS (Hamamatsu ORCA Flash4) camera. z-stack images were captured with axial step size of 0.5μm and an axial range of 6μm.

For membrane tubulation assay, the cells were treated with 100nM of cytochalasin D or DMSO in cell culture media for up to 40 min and fixed with cold methanol for 10 min. For quantification of cells with LTMs, z-stack images (axial step size of 0.3μm and axial range of 6μm) were captured with the SDCM using a 63X 1.3NA Zeiss oil objective and a sCMOS (Hamamatsu ORCA Flash4) camera and 12–15 different ROIs were analyzed for each experiment. For representative images, median filters were applied to the raw images using ImageJ software and quantification assay was performed using SlideBook software. For LTM imaging in zebrafish, single plane images of live cells on the surface of the yolk from 24 h post fertilization (hpf) embryos were captured every second for 30s using the 63x 1.3 NA oil objective on the SDCM.

For LTM colocalization studies, cells were grown on glass bottom plates (#1.5), treated with CytoD for indicated times, fixed as mentioned above and imaged using an Elyra7 microscope (Carl Zeiss Jena, Germany) with a Plan-Apochromat 63X 1.4NA oil Zeiss objective and a sCMOS camera (Hamamatsu). Image stacks were captured in Lattice SIM mode with a step size of 0.3μm for a axial range of 7μm and reconstructed using the SIM^2^ algorithm in a 3D leap mode to achieve a resolution of 60nm and a axial step size of 0.1μm. For live imaging LTMs or vesicles, cells were imaged as described above at 37C with 5% CO_2_ using a Plan-Apochromat 40X 1.4NA oil Zeiss objective in Apotome mode for single z-planes, two channels captured simultaneously, and reconstructed using the Burst mode and SIM^2^ algorithm as previously described.^48^ Cells were illuminated with 488nm OPSL laser and 561nm OPSL laser simultaneously using two Hamamatsu ORCA-Fusion sCMOS cameras. For Elyra7 calibration and alignment, a slide with tetra Speck beads (Zeiss) was used. Alignment parameters were determined, and the image processing was performed with Zeiss Zen Black software. The histograms of the processed images were adjusted using ImageJ without the use of additional filters. Colocalization maps were generated using ImageJ Colocalization threshold Plugin. Colocalization coefficients (Pearson’s Correlation coefficient) were calculated using MATLAB for three tubulated regions of each cell and five cells per experiment.

Super resolution imaging of the cilia was performed using the Elyra7 microscope. Cells were fixed with PFA 12 h after starvation as described above. The same image acquisition procedure was followed as described above for imaging LTMs. For ciliogenesis, z-stacks of two channel simultaneous images were captured at multiple positions every 10 min after 3 h starvation with a Plan-Apochromat 40X 1.4NA oil Zeiss objective in Apotome mode (axial range of 7μm with a step size of 0.3μm), and images were reconstructed using 3D Leap mode and SIM^2^ algorithm. The intensity line profiles were generated using ImageJ as previously described.^30^

For time-lapse mCherry-Rab8a membrane loading experiments, cells were treated with 1ug/ml doxycycline and imaged using a ZEISS SP5 laser scanning confocal at 37C in 5% CO_2_ with a 100X 1.47NA Zeiss oil objective at the times indicated.^3^ Slidebook was used for image acquisition and analysis, and IMARIS was used to identify and measure fluorescence intensity in tBFP-Rab11b positive structures with volumes greater than or equal to 0.37μm^3^ (1 voxel).

#### Immunoblotting

Western blotting analysis was performed as previously described.^3^ Briefly, cells were lysed using low salt Triton buffer (30mM Tris-HCL, 75mM NaCl, 10% Glycerol, 1% Triton X-100) and protease and phosphatase inhibitor cocktail (#78441, Thermo Fisher Scientific). For zebrafish, injected embryos were harvested at 24 hpf and homogenized in lysis buffer (20mM Tris, 137mM NaCl, 10% Glycerol and 1% Triton X-100) supplemented with protease and phosphatase inhibitor cocktail (#78441, Thermo Fisher Scientific). Lysates were centrifuged for 10 min at 14,0000 rpm and the supernatant was collected and sample buffer was added, followed by 5 min boiling at 95°C. Samples were separated using SDS-PAGE, transferred to nitrocellulose membrane (0.45μm) and probed with antibodies. Membranes were incubated with HRP conjugated secondary antibodies and imaged by chemiluminescence and iBright Imaging Systems (Thermo Fisher Scientific), respectively, according to the manufacturer’s protocol. Protein expression levels were compared using ImageJ software as previously reported.^46^ Briefly, for all the cell lines (RPE tRFP-Rab8a, RPE tRFP-Rab8a+GFP-Rab11a, RPE tRFP-Rab8a+GFP-Rabin8, RPE tRFP-Rab8a+GFP-Rabin8+miRFP670-Rab11a and RPE tRFP-Rab8a+GFP-Rabin8+miRFP670-Rab14) the exogenous and endogenous protein levels were determined by direct comparison to the endogenous levels of RPE WT cells for +/−Dox treated conditions. The protein levels was normalized to the GAPDH level.

#### Zebrafish micro-injection

Zebrafish experimental procedures were performed in accordance with protocols (ASP#23–416) approved by the animal care & use committee of the National Cancer Institute at Frederick in compliance with the Association for Assessment and Accreditation of Laboratory Animal Care (AAALAC) guidelines. One cell stage embryos from AB zebrafish were micro-injected with 250 μM Ctrl morpholino (MO) obtained from Gene Tools (Ctrl MO 5′- CCTCTTACCTCAGTTACAATTTAT -3′) or the translation blocking *rabin8* MO (^3^, 5′- TCTTCATACAGCACTCTGAGAAAAC -3′). capped mRNAs were *in vitro* transcribed following the mMessage mMachine SP6 Transcription Kit protocol (ThermoFisher, AM1340). To test the effects of Rabin8 mutants Capped mRNAs of mNeonGreen-Rab8a and mCherry-Rab11a along with untagged Rabin8 or Rabin8E192A were micro-injected at 250pg.

### QUANTIFICATION AND STATISTICAL ANALYSIS

Statistical analysis (two tailed unpaired student’s t test) as reported were performed using GraphPad Prism for Windows or Mac. Means ± SEM are specified in the figure legends. In the figure legends, indicated n is the number of cells or zebrafish embryos counted. *p* values have been indicated as follows **** > 0.00001, *** > 0.0001, ** > 0.01, * > 0.05.

## Supplementary Material

1

2

3

## Figures and Tables

**Table:** KEY RESOURCES TABLE

REAGENT or RESOURCE	SOURCE	IDENTIFIER
Antibodies		
Rab8	BD Biosciences	Cat# 610844; RRID: AB_398164
Rab8a	Proteintech	Cat# 55296-1-AP; RRID: AB_10858398
GFP	Miltenyi Biotec	Cat# 120 002 105; RRID: AB_247003
Actin	Sigma	Cat# A3854
^Ac^tubulin	Sigma	Cat# T6793; RRID: AB_477585
Pericentrin	Novus Biologicals	Cat# NB10061071
miRFP703	Invitrogen	Cat# PA5-109200; RRID: AB_2854611
tRFP	Evrogen	Cat# AB233; RRID: AB_2571743
Rab11a	Life Technologies	Cat# 715300; RRID: AB_2533987
GAPDH	Proteintech	Cat# HRP-60004; RRID: AB_2737588
Rabin8	Proteintech	Cat# 12321-1-AP; RRID: AB_2177510
c7orf43	Novus Biologicals	Cat# NBP1-83808
FIP3	Proteintech	Cat# 25843-1-A-P; RRID: AB_2880264
EHD1	Abcam	Cat# ab109311; RRID: AB_10859459
WDR44	Bethyl laboratories	Cat# A301-440A
CEP164	In House^46^	
HRP conjugated anti-Rabbit	Cytiva	Cat# NA934V
HRP conjugated anti-Mouse	Cytiva	NA931V
Alexa 647 anti-chicken	Jackson Immunoresearch Laboratories	Cat# 703-605-155; RRID: AB_2340379
DyLight405 anti-chicken	Jackson Immunoresearch Laboratories	Cat# 703-475-155; RRID: AB_2340373
Alexa 488 anti-Mouse	Jackson Immunoresearch Laboratories	Cat# 715-545-151; RRID: AB_2341099
Alexa 647 anti-Mouse	Jackson Immunoresearch Laboratories	Cat# 715-605-151; RRID: AB_2340863
DyLight405 anti-Rabbit	Jackson Immunoresearch Laboratories	Cat# 711-475-152; RRID: AB_2340616
Bacterial and virus strains		
Top 10	Life Technologies Corp	C404003
DH5α	Life Technologies Corp	1825801
Stabl3	Life Technologies Corp	C737303
Chemicals, peptides, and recombinant proteins		
Cytochalasin D	Tocris Bioscience	1233
Doxycycline monohydrate	Sigma	D1822
Lipofectamine 3000	Life Technologies	L3000001
Fugene6	Promega	E2691
Polyjet	Signagen Laboratories	SL100688
Lipofectamine RNAiMAX	Life Technologies	13778150
Puromycin	Invitrogen	A1113803
Blasticidin	Santa Cruz biotechnology	sc-495389
Neomycin	Invitrogen	10131035
Protease and phosphatase inhibitor cocktail	Thermo Fisher Scientific	78441
Phusion polymerase	Thermo Fisher Scientific	F632S
BSA	Sigma	A2153-100G
Triton X-100	Sigma	T8787-250ML
Glycerol	Sigma	G9012-1L
PFA	Electron Microscopy Sciences	RT15714
LR clonase II	Thermo Fisher Scientific	11791100
BP Clonase II	Thermo Fisher Scientific	11789100
LR Clonase II+	Invitrogen	12538120
ECL	Thermo Fisher Scientific	32106
ECL Super signal	Thermo Fisher Scientific	34577
Experimental models: Cell lines		
Human Retinal Pigment Epithelial cells hTERT-RPE1 (RPE)	ATCC	CRL-4000
293T	ATCC	CRL-3216
RPE tRFP-Rab8a	This paper	
RPE tRFP-Rab8a+GFP-Rab11a	This paper	
RPE tRFP-Rab8a+GFP-Rabin8	This paper	
RPE tRFP-Rab8a+GFP-Rabin8E192A	This paper	
RPE tRFP-Rab8a+GFP-Rabin8F201A	This paper	
RPE tRFP-Rab8a+GFP-Rabin8+miRFP670Rab11a	This paper	
RPE tRFP-Rab8a+GFP-Rabin8+miRFP670Rab14	This paper	
RPE GFP-Rabin8	Westlake et al.^3^	
RPE GFP-Rabin8+mCherry-Rab8a	This paper	
Experimental models: Organisms/strains		
Zebrafish/AB	ZIRC	ZL1
Oligonucleotides		
siRNA targeting sequence: siGFP ACAUGAAGCAGCACGACUUUU	Dharmacon	Custom/OTP
siRNA targeting sequence: sitRFP#1 GCACCCAGACCAUGAGAAUUU	Dharmacon	Custom/OTP
siRNA targeting sequence: sitRFP#2 AGGGCGAAGAGCUGAUUAA UU	Dharmacon	Custom/OTP
siRNA targeting sequence: siRabin8 CAAGAAAGGACAACTATAA	Dharmacon	Custom/OTP
siRNA targeting sequence: siC7orf43 #1 ACAAGATTGCCAAGCGCGA	Dharmacon Cuenca et al.^33^	J-016464- 19
siRNA targeting sequence: siC7orf43 #2 AGAGGGTGGTGATGGCTAA	Dharmacon Cuenca et al.^33^	Custom/OTP
siRNA targeting sequence: siFIP3 AGGCCAACAUUGAGCGUCU	Dharmacon Walia et al.^16^	J-021079-20
siRNA targeting sequence: siWDR44 #1 GUAUAAGGGUUACGUCAAU	Dharmacon Walia et al.^16^	J-018913-09
siRNA targeting sequence: siWDR44 #2 CUUCAGAAAGUACGGUUAA	Dharmacon Walia et al.^16^	J-018913-10
siRNA targeting sequence: siEHD1 UGAUGGUGAUGGUGCGGCAUU	Dharmacon Lu et al.^30^	Custom/OTP
siRNA targeting sequence: siRab11a GCAAACAAUGUGGUUCCUAUUU	Dharmacon Westlake et al.^3^	J-004726-07
siRNA targeting sequence: siRab11b GGGACGACGAGUACGACUAUU	Dharmacon Westlake et al.^3^	Custom/OTP
Morpholino: *rabin8*MO sequence: TCTTCATACAGCACTCTGAGAAAAC	Gene Tools Westlake et al.^3^	Custom
Recombinant DNA		
psPAX2	Addgene	12260
PMD2.G	Addgene	12259
pTagBFP-N	Evrogen	FP172
mNeonGreen	Addgene	189976
pENTR-Rab7	Thermo	IOH40569
pENTR-Rab14	Thermo	IOH6559
pENTR Rab11a	Westlake et al.^3^	
pENTR Rab11b	Westlake et al.^3^	
pENTR Rabin8	Westlake et al.^3^	
pENTR Rab8a	Westlake et al.^3^	
pHUSH-mCherry	Westlake et al.^3^	
pHUSH-tRFP (CMV promoter)	Westlake et al.^3^, Lu et al.^30^	
pDONR221	Invitrogen	12536017
pDONR221 P5,P2	Invitrogen	12537100
pDONR221 P1-P5r	Invitrogen	12537100
pDONR235	Kind gift from Dominic Esposito	
pHUSH tRFP-Rab8a	This paper	
pDONR Rab5a	This paper	
pDONR Rab6a	This paper	
pDONR Rabin8 E192A	This paper	
pDONR Rabin8 F201A	This paper	
mCherry-Rab8a	This paper	
mCherry-Rab7	This paper	
mCherry-Rab6a	This paper	
BFP-Rab11b	This paper	
BFP-Rab14	This paper	
pENTR Rab11aS25N	Walia et al.^16^	
pENTR Rab11bS25N	Walia et al.^16^	
pENTR Rab5aS34N	This paper	
pENTR Rab14S25N	This paper	
pDest53	Invitrogen	12288015
GFP-Rab11aSN	This paper	
GFP-Rab11bSN	This paper	
GFP-Rab5aSN	This paper	
GFP-Rab14SN	This paper	
pENTR Rab11aQ67L	Walia et al.^16^	
pENTR Rab11bQ67L	Walia et al.^16^	
pENTR Rab14Q70L	This paper	
pDEST tRFP	Westlake et al.^3^	
tRFP-Rab11a	This paper	
tRFP-Rab11aQL	This paper	
tRFP-Rab11b	This paper	
tRFP-Rab11bQL	This paper	
tRFP-Rab14	This paper	
tRFP-Rab14QL	This paper	
pCS2+	Addgene	1000000107
pCS2-GFP	Addgene	86723
mNeonGreen-Rab8a	This paper	
mCherry-Rab11a	This paper	
pPGK	Addgene	197201
miRFP670	Addgene	197201
miRFP670(5,1)	This paper	
EF1α	Addgene	162975
GFP	Addgene	162970
pDEST686	Addgene	161889
pDEST658	Addgene	161882
pDEST686-pGK-miRFP670 Rab11	This paper	
pDEST686-pGK-miRFP670 Rab14	This paper	
pDEST658-EF1α GFP-Rab11a	This paper	
pDEST658-EF1α GFP-Rabin8	This paper	
pDEST658-EF1α GFP-Rabin8E192A	This paper	
pDEST658-EF1α GFP-Rabin8F201A	This paper	
Software and algorithms		
Fiji/ImageJ		https://imagej.net/software/fiji/
GraphPad Prism 9		https://www.graphpad.com/
MATLAB version 2022b		https://www.mathworks.com/

## Highlights

- Rab8 promotes Rab11 ciliary targeting supportive of a Rab cascade membrane conversion model
- Rab11-Rab8 cascade dynamics, mediated by Rabin8, can be visualized on membrane tubules
- Rab8 membrane tubule localization requires trafficking factors needed for ciliogenesis
- Rab8 loading onto Rab11-Rabin8 membranes further supports a membrane conversion model
