# Supplementary material for: Rab11-Rab8 cascade dynamics in primary cilia and membrane tubules
Source: Cell Rep. Author manuscript; Available in PMC 2025 Apr 9. (PMC11980590; doi:10.1016/j.celrep.2024.114955)
Supplement: 1 [file NIHMS2038913-supplement-1.pdf]

Cell Reports, Volume *43*

Supplemental information

Rab11-Rab8 cascade dynamics in primary cilia and membrane tubules

Ipsita Saha, Christine Insinna, and Christopher J. Westlake

Figure S1

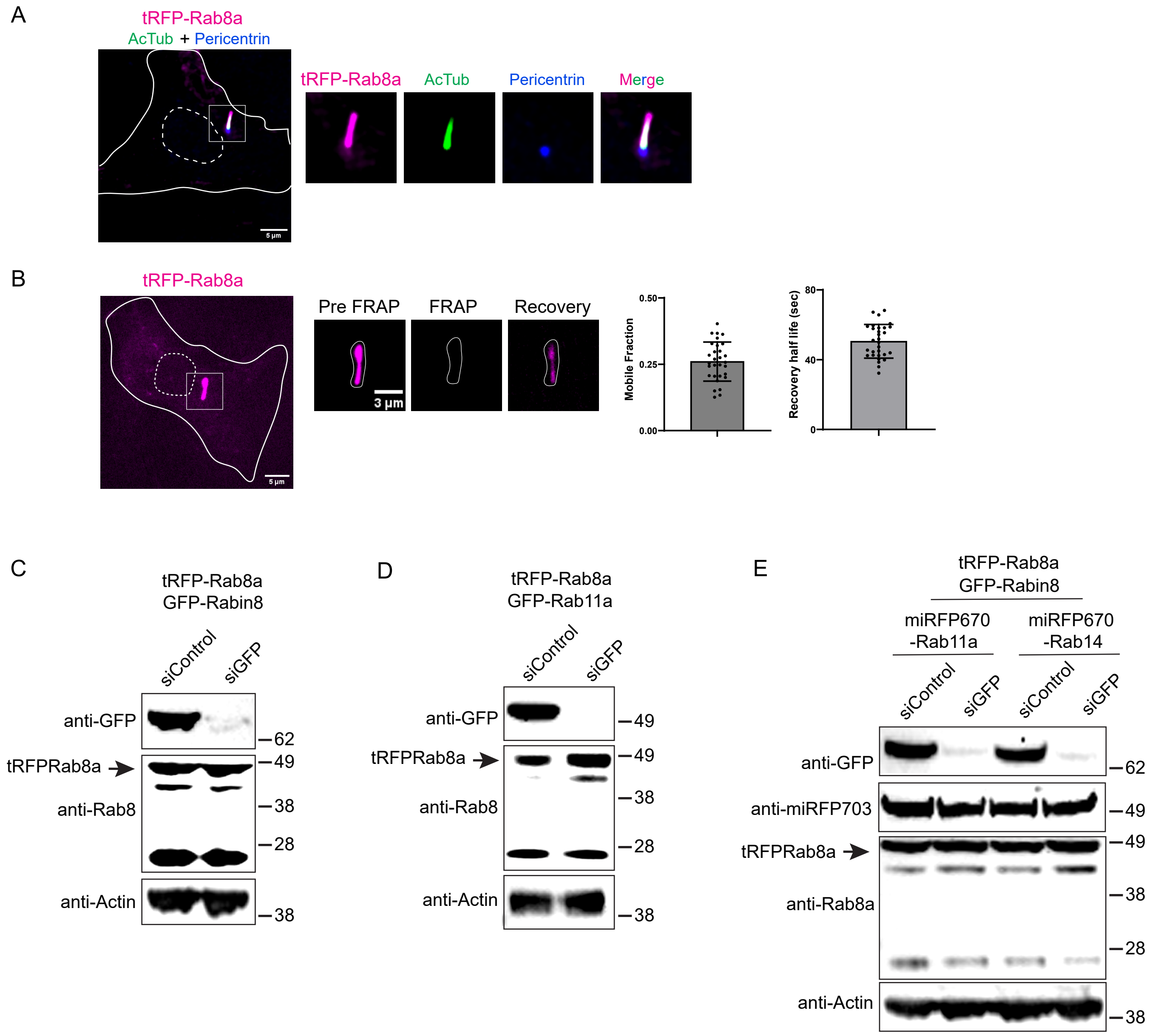

Figure S1: tRFP-Rab8 FRAP recovery and RNAi protein expression analysis

(A) Representative image of a RPE tRFP-Rab8a cell, treated with Dox for 24 h, fixed post 12 h of serum starvation, and stained with ^{Ac}Tub and Pericentrin. (B) Quantification of FRAP recovery of ciliary tRFP-Rab8a in cells described and treated as in (A). FRAP was performed as described in Figure 1. Representative image from 10 cells analyzed and plots show mean ± SD from n=3 experiments. Two-tailed unpaired Student's t-test. ****P < 0.00001. Scale bar: 3μm (C, D) Immunoblot analysis of lysates from cells as described in Figure 1C and Figure 1D and stained with GFP, Rab8 and actin antibodies. (E) Immunoblot analysis of lysates from RPE cells as described in Figure 1E and stained with GFP, miRFP703, Rab8a, and actin antibodies.

Figure S2

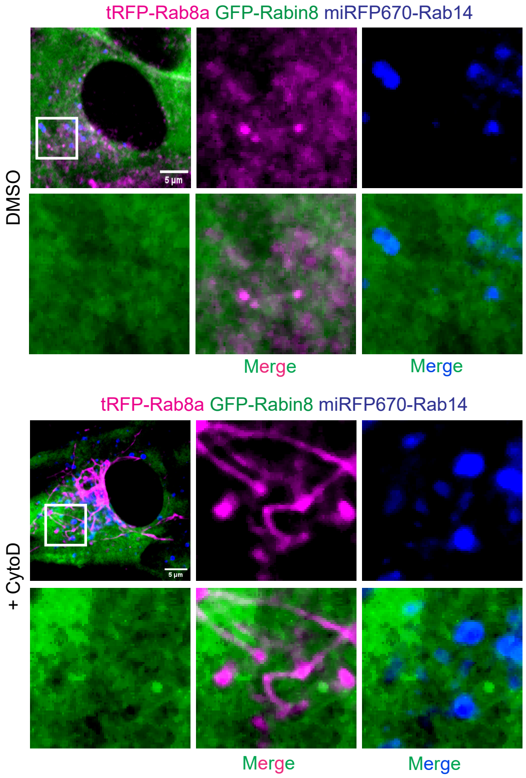

Figure S2: Rab14 does not colocalize with Rab8-positive LTMs

Localization analysis of Rab14 on Rab8 LTMs. Representative images of a single xy-plane from a z-stack of RPE tRFP-Rab8a+GFP-Rabin8+miRFP670-Rab14 cells, treated with Dox for 24 h followed by DMSO or CytoD for 30 min and imaged on a SDCM. Scale bar: 5µm.

Figure S3

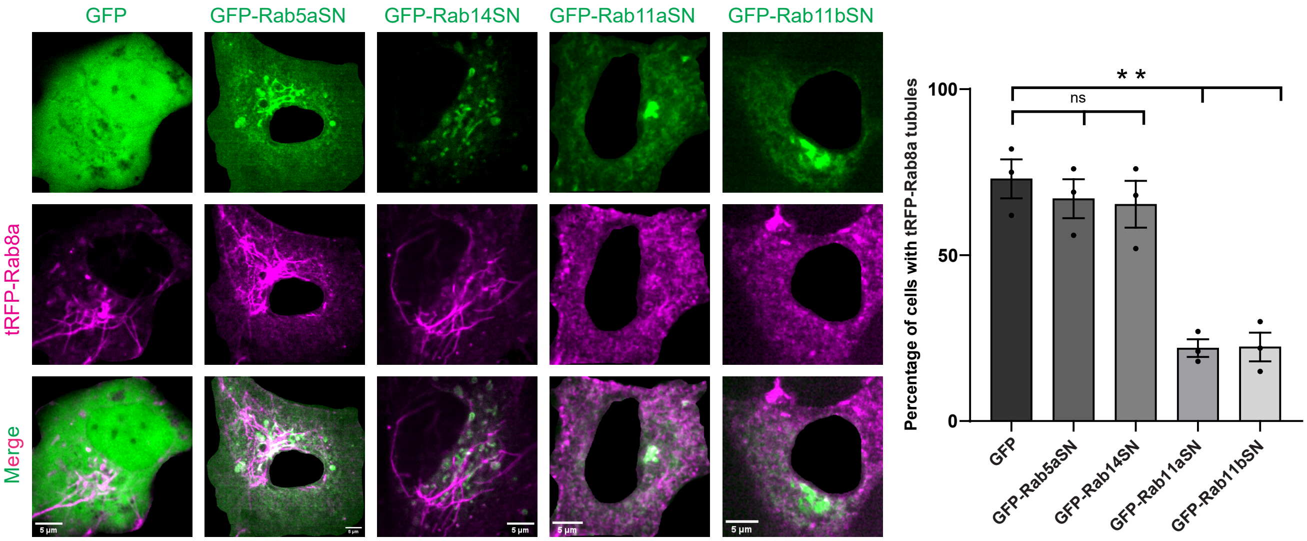

Figure S3: tRFP-Rab8a LTM association is blocked by Rab11 dominant negative expression

Effects of Rab dominant negative mutant expression on Rab8 LTM formation. Left: Representative images of RPE tRFP-Rab8a cells transiently transfected with GFP control or GFP-Rab dominant-negative proteins and treated with Dox for 24 h followed by CytoD for 30 min. Cells were imaged 48 h post transfection. Images shown are from a single xy-plane of a *z*-stack captured by SDCM. Right: Quantification of cells with tRFP-Rab8a LTMs. Mean ± SEM for ~100 cells from n=3 experiments; **p<0.001. Scale bar: 5µm.

Figure S4

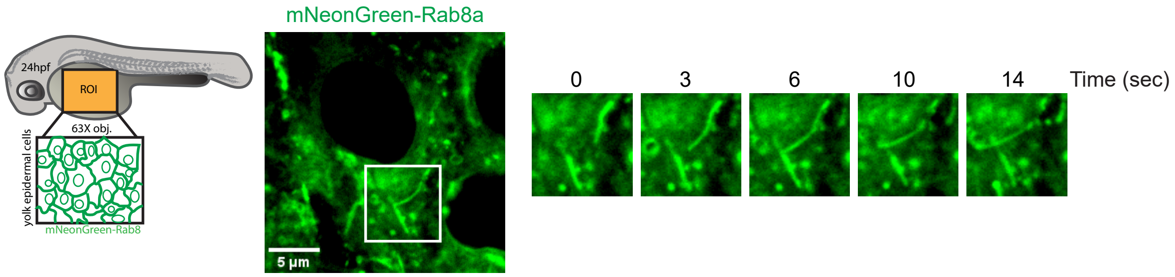

Figure S4: Dynamic Rab8 LTMs in zebrafish

Visualization of Rab8 LTMs in zebrafish. Left: Model depicting the region of interest (ROI) imaged of the yolk sack from a 24 hpf zebrafish embryo injected with mNeonGreen-Rab8a at the one cell stage. Right: Representative single xy-plane image of zebrafish yolk cells ectopically expressing mNeonGreen-Rab8a imaged as shown in model by live SDCM every 1 s.

Figure S5

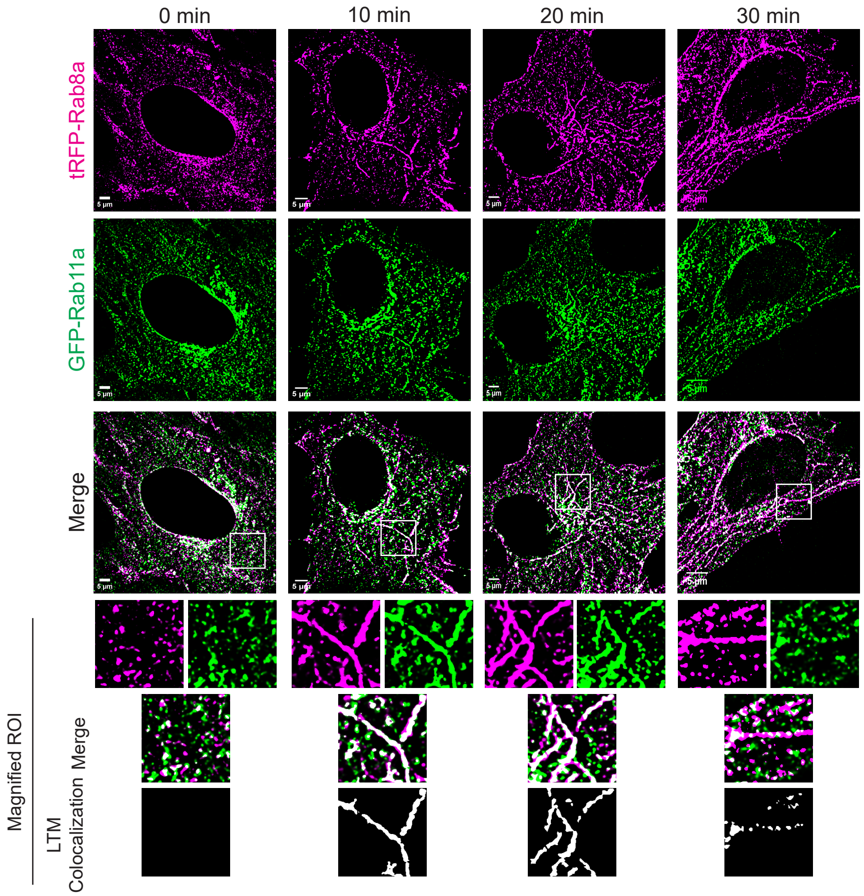

Figure S5: Dynamics of Rab11 and Rab8 on CytoD stimulated LTMs

Representative SIM^2 images of fixed RPE cells as described in Figure 4A treated with CytoD over time. Images are from a single xy-plane of a z-stack image captured onElyra7 microscope. tRFP-Rab8a+GFP-Rab11a colocalization on LTMs from the magnified ROIs are shown below. Scale bar: 5µm.

Figure S6

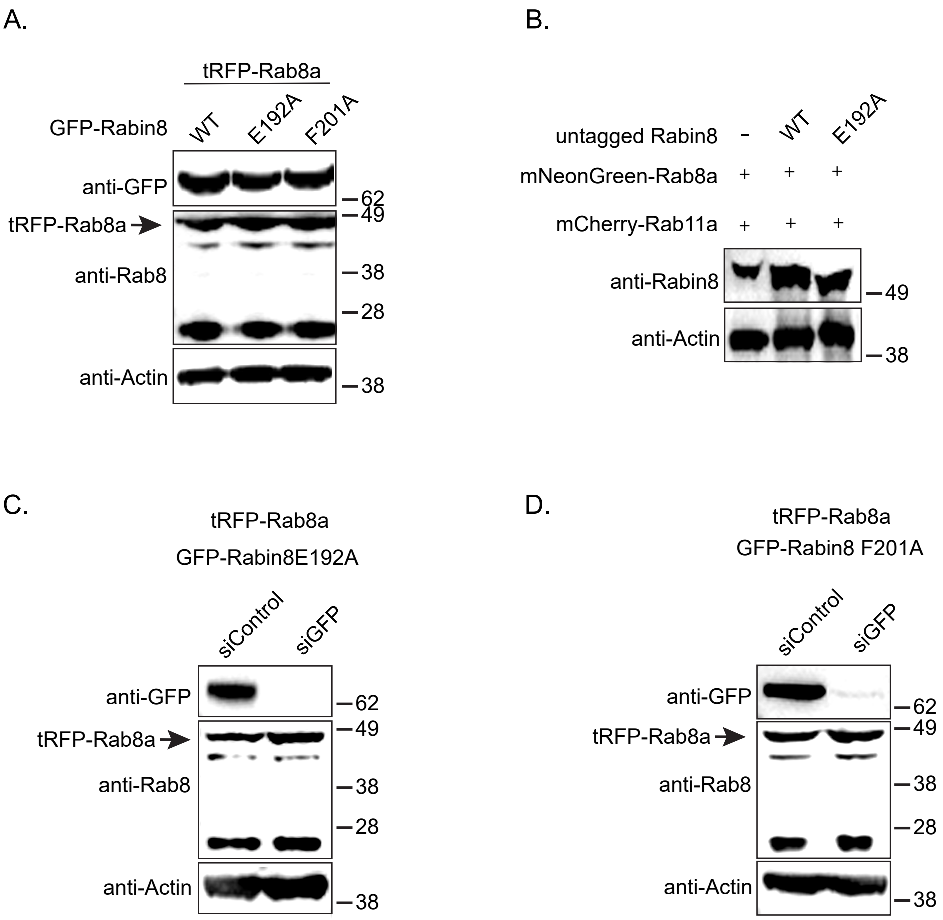

Figure S6: Rabin8 WT and mutants exogenous expression analysis

(A) Immunoblot analysis of lysates from RPE tRFP-Rab8a+GFP-Rabin8, tRFP-Rab8a+GFP-Rabin8 E192A and tRFP-Rab8a+GFP-Rabin8 F201A cells treated with Dox for 24 h and probed with GFP, Rab8 and actin antibodies. (B) Immunoblot analysis of zebrafish embryos as described in Figure 5B and stained with Rabin8 and actin antibodies. (C, D) Immunoblot analysis of lysates from RPE tRFP-Rab8a cells expressing Rabin8 mutants treated with siControl and siGFP as described in Figure 5D and stained with GFP, Rab8, and actin antibodies.
